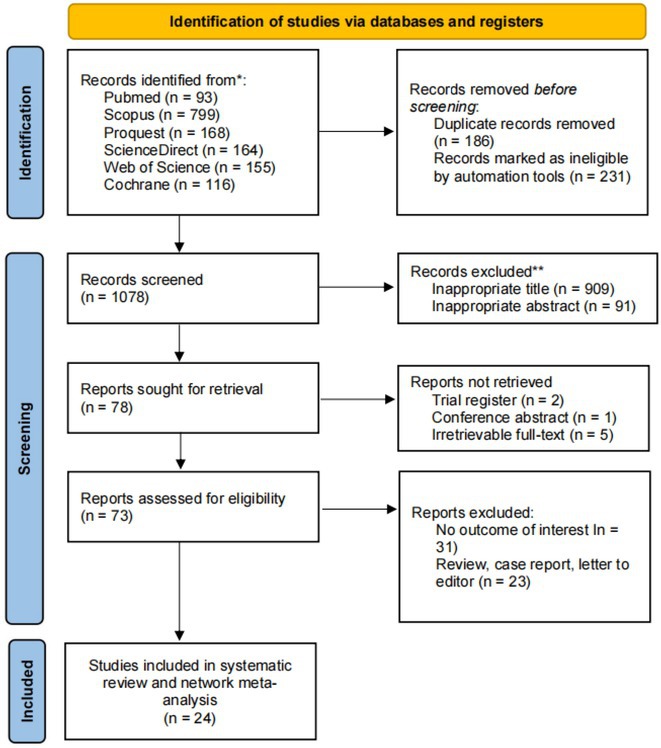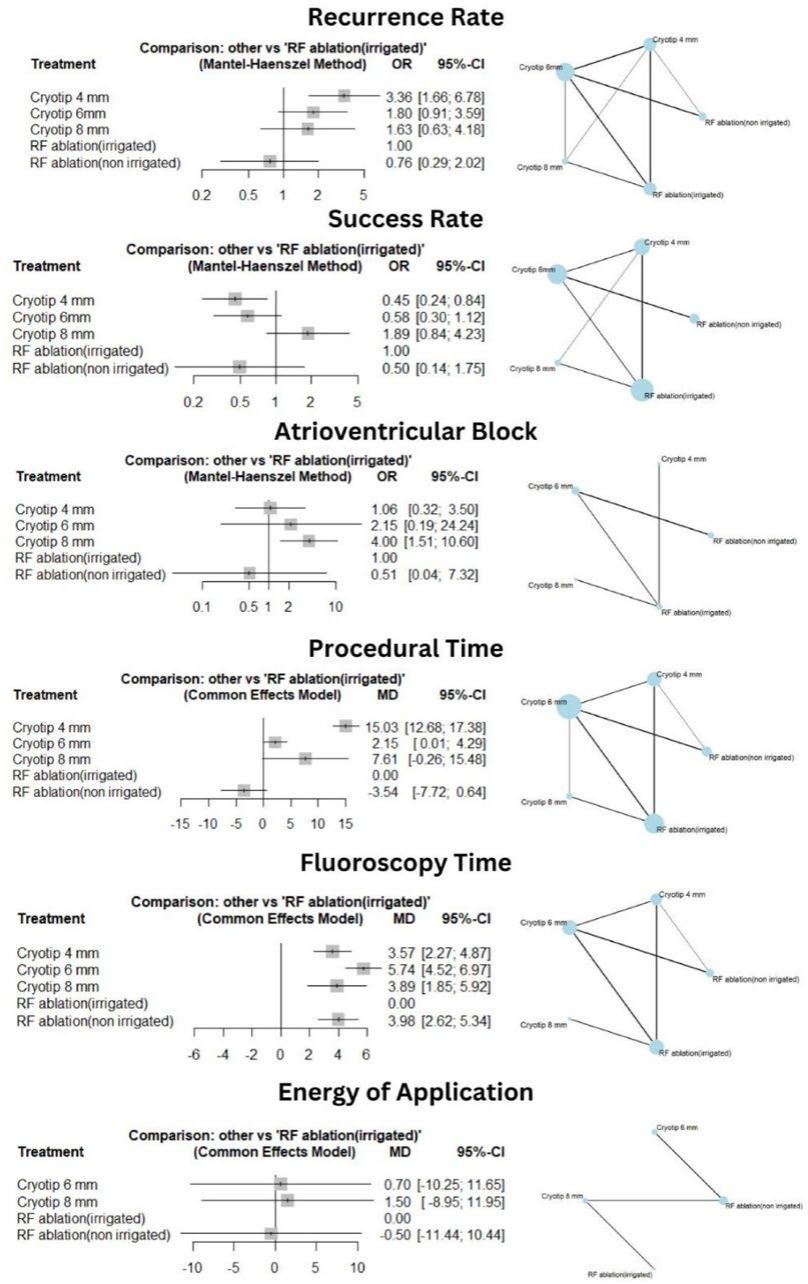# Best Poster Abstracts for the 17th Asia Pacific Heart Rhythm Society (APHRS) Scientific Sessions

**DOI:** 10.1002/joa3.70002

**Published:** 2025-03-14

## 1 DELIBERATE ENDOCARDIAL BREACHING TO PREVENT ENTANGLEMENT IN CONDUCTION SYSTEM PACING LEADS IN AN EX‐VIVO MODEL

### 1.1 **DARIUS CHAPMAN**, CAMPBELL STRONG, IVAYLO TONCHEV, PRABHPREET KAUR, ANAND GANESAN

#### 1.1.1 Flinders University, Bedford Park, Australia

**Introduction:** Failure to deploy leads due to entanglement or drill effect in conduction system pacing (CSP) can lead to deployment failure or damage. We investigated whether deliberate endocardial breaching (EB) prior to CSP lead placement reduced complications by mitigating interactions between lead and endocardium.

**Methods:** An endocardial breaching catheter was used to create a small (~2mm x 2mm) endocardial breach at the deployment site (intervention group). Controls received no breach. In an ex‐vivo ovine heart model, 240 CSP leads (120 lumen‐less Medtronic 3830, 120 stylet‐driven Biotronik Solia) were deployed at varying angles (45°, 60°, and 90°) with a controlled back‐support. Primary endpoints were the incidence of adverse events (lead entanglement or drill effect) and number of rotations to achieve a 10mm depth.

**Results:** 240 CSP leads were deployed in random order to EB or control groups. Deliberate EB significantly (p<0.001) reduced entanglement and drill effect for both lumen‐less leads (LLL) (ctrl = 30/60, 50%; EB = 4/60, 6.7%) and stylet‐driven leads (SDL) (Ctrl = 7/60,12%; EB = 0/60, 0%). For LLL, the odds ratio for adverse events with EB was 0.074 (95% CI 0.02‐0.23). For SDL, events were reduced in EB to 0/60 (0%). Additionally, EB significantly reduced the number of rotations needed to achieve a 10mm depth for both lead types (p<0.001), with reductions of 19.57 rotations (95% CI 14.55‐24.58) for LLL and 5.77 rotations (95% CI 3.94‐7.59) for SDL. This benefit was consistent across all lead‐to‐septum angles.

**Conclusions:** Deliberate endocardial breaching before CSP lead deployment significantly reduces adverse lead events and the number of rotations required to achieve target depth in an ex‐vivo ovine model. This study demonstrates a key mechanism of CSP‐related complications, and how the elastic endocardium can be modified procedurally via deliberate endocardial breaching to improve lead deployment.

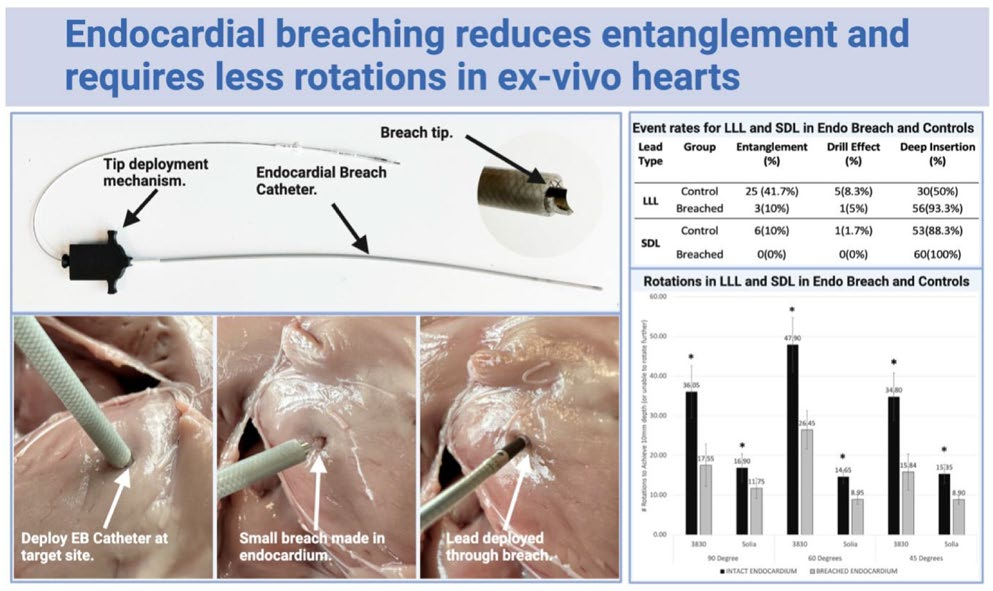

## 2 ONE‐YEAR ELECTRICAL PERFORMANCE OF THE FIRST DUAL‐CHAMBER LEADLESS PACEMAKER

### 2.1 REINOUD KNOPS^1^, **RAHUL DOSHI** ^2^, JAMES E. IP^3^, PASCAL DEFAYE^4^, DEREK V. EXNER^5^, GERHARD HINDRICKS^6^, MORIO SHODA^7^, MARIA G. BONGIORNI^8^, ROBERT CANBY^9^, PETR NEUŽIL^10^, THOMAS CALLAHAN^11^, SRI SUNDARAM^12^, NIMA BADIE^13^, VIVEK REDDY^14^

#### 2.1.1 ^1^AMC Amsterdam, Amsterdam, Netherlands,^2^HonorHealth Cardiac Arrhythmia Group, Scottsdale, AZ,^3^Weill Cornell Medicine, New York, NY,^4^Centre Hospitalier Universitaire Grenoble Alpes, Grenoble, France,^5^Foothills Medical Centre, Calgary, AB, Canada,^6^Deutsches Herzzentrum der Charite, Berlin, Germany,^7^Tokyo Women's Medical University, Tokyo, Japan,^8^San Rossore Private Hospital and Medical Center, Pisa, Italy,^9^Texas Cardiac Arrhythmia Institute, Austin, TX,^10^Na Homolce Hospital, Prague, Czech Republic,^11^Cleveland Clinic Foundation, Cleveland, OH,^12^South Denver Cardiology, Littleton, CO,^13^Abbott, Sunnyvale, CA,^14^Mount Sinai Fuster Heart Hospital, New York, NY

**Introduction:** The first dual‐chamber leadless pacemaker (LP) includes helix‐fixation atrial and ventricular LPs (ALP, VLP) with implant‐to‐implant (i2i) communication to maintain atrioventricular synchrony. The safety and overall performance have been demonstrated in a clinical trial, but the long‐term electrical performance has not been reported.

**Methods:** ALP and VLP electrical performance metrics were collected as part of a prospective, international clinical trial (NCT05252702). Patients indicated for dual‐chamber pacing were implanted with Aveir DR (Abbott). Pacing capture threshold (PCT) at 0.4 ms pulse width, sensed amplitude, pacing impedance, and i2i communication success were collected from implant to 12 months (12M).

**Results:** Of 500 patients enrolled, 12M electrical measurements were collected in 290 patients with *de novo* implants (63% male; 70 ± 14 years; 80 ± 20 kg; 63% sinus node dysfunction, 13% complete AV block), excluding 3 ALPs and 4 VLPs revised due to capture issues. PCTs at implant (ALP 2.5 ± 1.5 V, VLP 0.8 ± 0.7 V) improved significantly by 1M post‐implant and remained stable through 12M (ALP 0.9 ± 0.7 V, VLP 0.7 ± 0.5 V). At 12M, PCT > 3.0V in 2% of ALPs and 1% of VLPs were observed. Sensed amplitudes at implant (ALP 1.8 ± 1.2 mV, VLP 9.0 ± 4.0 mV) also improved significantly by 1M and remained stable through 12M (ALP 3.4 ± 1.8 mV, VLP 11.6 ± 4.2 mV). Impedances at implant (ALP 334 ± 66 Ω, VLP 791 ± 349 Ω) reduced by 3M and remained stable through 12M (ALP 310 ± 47 Ω, VLP 600 ± 146 Ω). Electrical metrics at 12M did not significantly differ across ALP or VLP implant locations. ALP‐to‐VLP and VLP‐to‐ALP i2i communication was successful in 91 ± 12% and 89 ± 15% of beats at 12M. Phrenic nerve stimulation was noted in 0.7% of patients.

**Conclusions:** The Aveir DR leadless pacemaker demonstrated stable, effective electrical performance throughout the 12‐month post‐implant evaluation period.

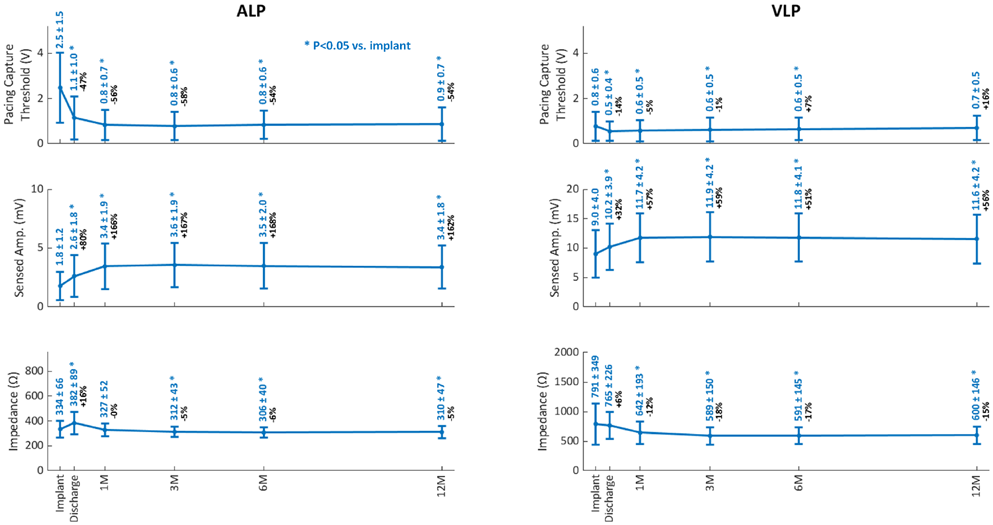

## 3 LEFT VENTRICULAR ELECTRICAL DELAY PREDICTS RESPONSE TO LEADLESS CARDIAC RESYNCHRONISATION THERAPY

### 3.1 **MARK ELLIOTT** ^1^, NADEEV WIJESURIYA^1^, VISHAL MEHTA^1^, FELICITY DE VERE^1^, SANDRA HOWELL^1^, NILANKA MANNAKKARA^1^, BALDEEP SIDHU^1^, PAOLO BOSCO^2^, PRASHANTHAN SANDERS^3^, JAGMEET P SINGH^4^, MARY NORINE WALSH^5^, STEVEN NIEDERER^6^, CHRISTOPHER ALDO RINALDI^1^

#### 3.1.1 ^1^Kings college London, London, United Kingdom,^2^Guys and St Thomas' NHS Foundation Trust, London, United Kingdom,^3^University of Adelaide, Adelaide, Australia,^4^Massachusetts General Hospital, Harvard Medical School, Boston, MA,^5^Ascension St. Vincent Heart Center, Indianapolis, IN,^6^National Heart and Lung Institute, Imperial College London, London, United Kingdom

**Introduction:** Leadless left ventricular (LV) endocardial pacing is an emerging cardiac resynchronization therapy (CRT) technology. The predictors of response to leadless CRT are poorly understood. Targeting the LV pacing electrode to endocardial sites with increased electrical latency (Q‐LV) may produce higher response rates. We aimed to examine the association between Q‐LV and response to CRT delivered with the leadless WiSE‐CRT system.

**Methods:** A post‐hoc analysis of the SOLVE‐CRT trial (n=122) examined the relationship between pacing site Q‐LV with rate of LV end systolic volume (LVESV) reduction >15% at 6 months. Multivariable regression analysis (MRA), ROC analysis and analysis of variance by Q‐LV quartile was performed. A cardiomyopathy phenotype subgroup analysis was performed.

**Results:** The mean implant site Q‐LV was 125.6ms ± 27. The mean Q‐LV in LVESV non‐responders was 121ms ± 30 versus 131ms ± 23 in responders (p=0.05). MRA identified Q‐LV as an independent response predictor (p=0.05). Analysis by Q‐LV quartile (Figure 1) demonstrated a significant improvement in response rate in quartile 4 (longest Q‐LV, 64%) compared to quartile 1 (shortest Q‐LV, 28%), p<0.01. This association was primarily driven by strong Q‐LV‐response correlation in patients with ischaemic cardiomyopathy (p=0.004 in MRA). Analysis according to Q‐LV quartile in the ischaemic cohort demonstrated a significant difference in CRT response rate between groups (p=0.035) with significantly increased response in quartile 4 (67%) compared to both quartile 1 (19%, p=0.01) and quartile 2 (30%, p=0.04). No significant differences were observed between LV pacing electrode position and probability of response (p=0.81), or between electrode position and Q‐LV (p=0.3).

**Conclusions:** Increased Q‐LV at the LV endocardial pacing site was associated with improved reverse remodelling following leadless CRT. Individualised targeting of LV endocardial sites with high Q‐LV may deliver additional benefit compared to empirical LV electrode implantation.

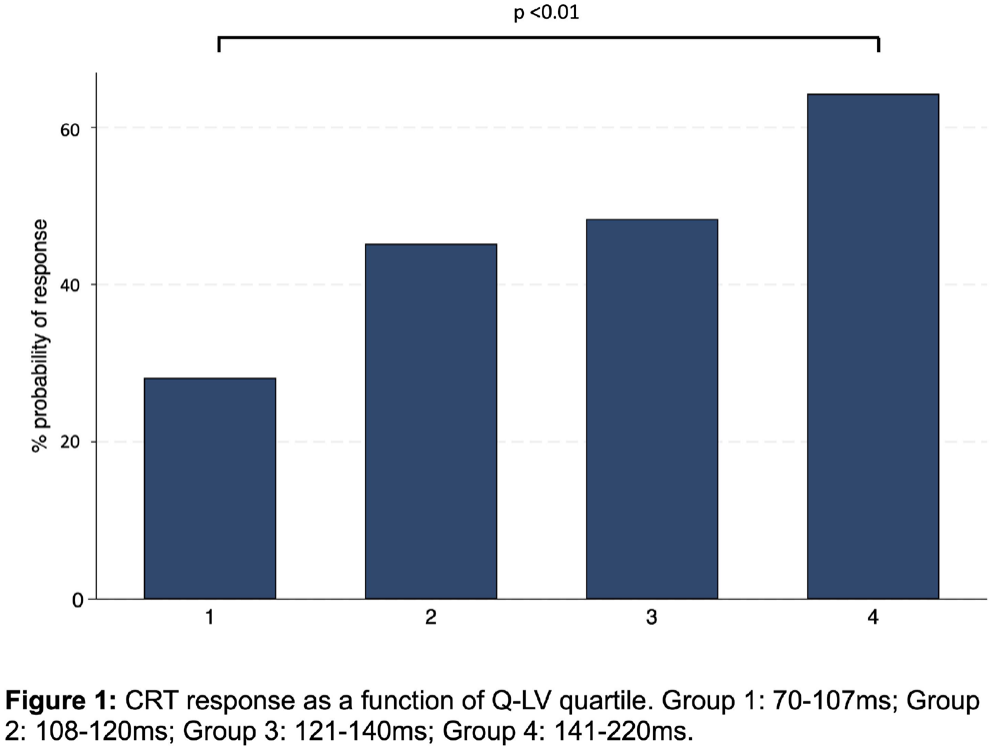

## 4 BEATBOT: A CUSTOMISED CHATBOT FOR CARDIAC IMPLANTABLE ELECTRONIC DEVICES (CIED) INQUIRIES

### 4.1 **PHOEBE GANIS** ^1^, TIMMY PHAM^1^, TORI STAMATOPOULOS^1^, IBRAHIM SHUGMAN^2^, TU HAO TRAN^1^, UPUL PREMAWARDHANA^2^

#### 4.1.1 ^1^Liverpool Hospital, Sydney, Australia,^2^Campbelltown Hospital, Sydney, Australia

**Introduction:** Cardiac Implantable Electronic Devices (CIED) interpretation is often complex. Large Language Models (LLM) chatbots are ubiquitous as tools for knowledge surveillance. Adoption in cardiology is impeded by the risk of misleading information. Use of Retrieval Augmented Generation (RAG) in a chatbot architecture can anchor the performance of an LLM by focusing it on a knowledge corpus to improve reliability. BeatBot is a customised chatbot for robust CIED inquiry.

**Methods:** BeatBot uses an open‐source platform Flowise AI^TM^ that consists of a document loader to ingest publicly available vendors manuals and programming tools (370 documents). A text splitter creates text chunks which are then embedded as vectors and deposited in a vector‐based storage. This RAG arrangement coupled with engineered prompts constrains the LLM to CIED literature. A Recall‐Oriented Understudy for Gisting Evaluation (ROUGE) score compared the outputs of Humans vs. ChatGPT 4o^TM^ (CGPT4) vs. Humans vs. BeatBot, where Humans (ground truth) denotes the ‘best’ collective response from 4 cardiac technologists. Varied topics were used to elicit responses which included Managed Ventricular Pacing, Asynchronous Pacing, Magnet Mode and Programming to avoid T wave oversensing.

**Results:** The responses from CGPT4 and BeatBot across various topics were compared using ROUGE scores. CGPT4 consistently showed lower precision, recall, and F1‐scores across all ROUGE‐1 (R1), ROUGE‐2 (R2), and ROUGE‐L (RL) metrics compared to BeatBot. BeatBot demonstrated significantly higher scores in both R1 and RL domains (precision: R1 0.46 vs. 0.12, RL 0.28 vs. 0.12; recall: R1 0.50 vs. 0.13, RL 0.31 vs. 0.13; F1‐score: R1 0.48 vs. 0.12, RL 0.29 vs. 0.13) suggesting a better overall performance. This indicates that BeatBot is more effective in generating accurate and comprehensive summaries compared to CGPT4.

**Conclusions:** BeatBot performs well compared to CPGT4, with more refinement it can be considered an educational platform and knowledge tool for cardiac technologists and medical staff in cardiology to assist in the management of patients with CIEDs.

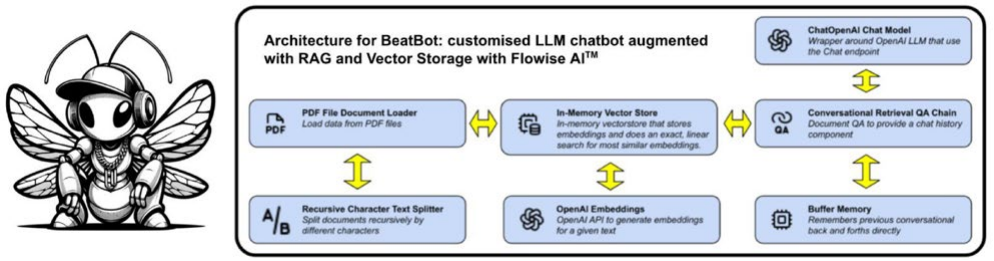

## 5 ACCURACY OF PERSONAL WEARABLE ECG DEVICES IN PACEMAKER PATIENTS

### 5.1 **PRIYA GARG**, CRAIG RIDDELL, MARINA FOWLER, KHANG LI LOOI, ROB DOUGHTY, MATTHEW O'CONNOR

#### 5.1.1 Department of Cardiology, Greenlane Cardiovascular Service, Auckland City Hospital, Auckland, New Zealand

**Introduction:** The use of wearable smart devices with electrocardiogram (ECG) recording capability is increasing. The aim of this study was to assess the accuracy of two selected wearables, the Apple Watch and AliveCor Kardia, in analysing heart rhythm and rate amongst patients with cardiac implantable electrical devices (CIEDs).

**Methods:** An observational study involving 100 patients with CIEDs was conducted at Auckland City Hospital between September 2023 and December 2023. During a routine CIED check, each patient's underlying rhythm and rate were confirmed and then compared against the interpretation displayed by the wearables. Following this, further recordings were obtained by the Apple Watch and AliveCor Kardia with the CIED programmed to bipolar then unipolar configuration and set at nominal and maximal outputs, respectively.

**Results:** The most common CIED was pacemaker (79%), followed by implantable defibrillator (19%), and cardiac resynchronisation therapy (2%). 7 cases of left bundle branch pacing were included. Bradycardia was the predominant indication for CIED implantation. Accuracy of rhythm interpretation was greater at bipolar settings compared to unipolar for both wearables. At nominal CIED output, rhythm interpretation was correct only half the time. When the CIED was set to maximal output, both the Apple Watch and AliveCor Kardia exhibited low accuracy in rhythm interpretation; particularly in the unipolar setting (see Figure 1). In most cases at maximal unipolar setting, both wearables either misinterpreted cardiac rhythm or failed to record an ECG. Compared to rhythm, heart rate was more accurately recorded by both wearables; this was most evident at the nominal bipolar setting (Apple Watch 84%; AliveCor Kardia 87%). Subgroup analysis of left bundle branch pacemakers demonstrated no difference in performance by either wearable.

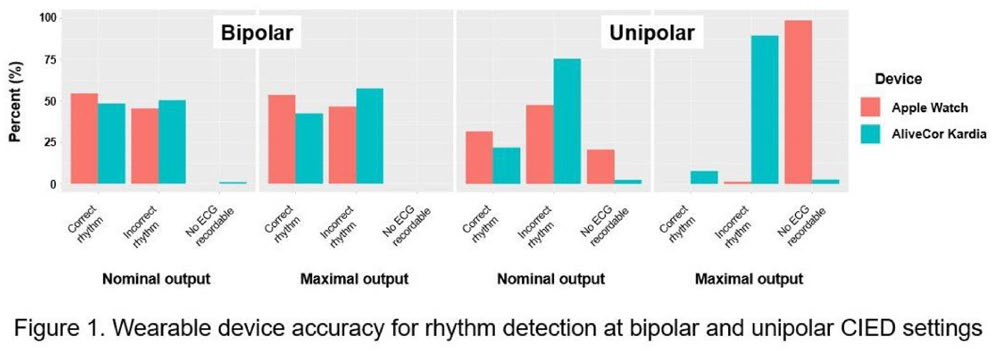

**Conclusions:** This study highlights that the Apple Watch and AliveCor Kardia wearable devices demonstrate poor utility in analysing heart rhythm and rate in patients with CIEDs. Further refinement of the detection algorithm is required before they can be reliably adopted into clinical practice in this important patient group.

## 6 SAFE AND RELIABLE LEADLESS PACEMAKER IMPLANTATION USING THE LATEST CONE‐BEAM CT SYSTEM

### 6.1 **YUICHI HORI**, HIDEYUKI AOKI, HIROTSUGU SATO, REIKO FUKUDA, SHIRO NAKAHARA

#### 6.1.1 Dokkyo Medical University Saitama Medical Center, Koshigaya, Japan

**Introduction:** The latest cone‐beam CT system; Dyna‐CT system can assist our leadless pacemaker (LP) implantation procedure, by importing the 3D information of the right ventricle (RV) accurately into the fluoroscopy view.

**Methods:** The utility of the Dyna‐CT system in LP implantation was investigated by comparing the procedure, with and without the assistance of the system. Thirty consecutive patients that underwent LP implantation with the assistance of Dyna‐CT system were enrolled, and was compared with the 30 previous cases without. Cone‐beam CT was taken during the RV angiography, and was imported to the fluoroscopy view. By having an assistance of the Dyna‐CT system, LP was implanted with having a navigational assistance inside the RV (left panel).

**Results:** There was no occurrence of cardiac perforation in all 60 patients. The radiation exposure for Cone‐beam CT was 147±33mGry. The time taken for the implantations was significantly shorter when having assistance from the system (p=0.04). By having locational information during the procedure, the LP was delivered safely and deployed precisely to the targeted aspect (center and right panel). The leadless pacemaker was implanted to the septum aspect in every case, which was confirmed from the CT taken after the procedure.

**Conclusions:** The use of the latest cone‐beam CT system contributes to the safe and shortening of procedure time for LP implantation. Performing a LP implantation with having a visual assistance may also lead to a reduction of fatal complications.

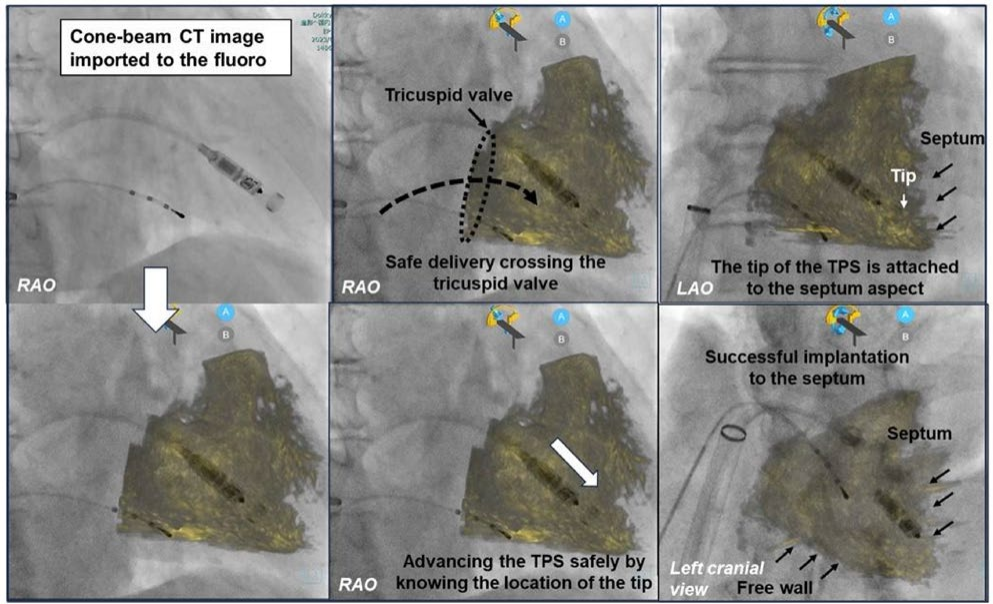

## 7 ALCOHOL FLUSHING SYNDROME AS A RISK FACTOR FOR ATRIAL FIBRILLATION RECURRENCE: ASSOCIATION BETWEEN *ALDH2* GENOTYPES AND ATRIAL FIBRILLATION RECURRENCE FOLLOWING CATHETER ABLATION

### 7.1 **TADASHI HOSHIYAMA** ^1^, KEIICHI ASHIKAGA^2^, KENJI MORIHISA^3^, MIWA ITO^2^, KENTARO ONIKI^4^, JUNJI SARUWATARI^4^, MASANOBU ISHII^1^, HISANORI KANAZAWA^1^, HITOSHI SUMI^1^, SHOZO KANEKO^1^, YUICHIRO TSURUTA^1^, KOHEI MATSUNAGA^1^, YUTA TSURUSAKI^1^, KENICHI TSUJITA^1^

#### 7.1.1 ^1^Kumamoto University Hospital, Kumamoto, Japan,^2^Miyazaki Medical Association Hospital, Miyazaki, Japan,^3^Kumamoto Chuo Hospital, Kumamoto, Japan,^4^Division of Pharmacology and Therapeutics, Graduate School of Pharmaceutical Sciences, Kumamoto University, Kumamoto, Japan

**Introduction:** Alcohol increases the risk of atrial fibrillation (AF) and is metabolized by aldehyde dehydrogenase 2 (ALDH2). Alcohol flushing syndrome, attributed to *ALDH2*‐deficiency increases AF developing when accompanied by habitual alcohol consumption. We investigated the association between *ALDH2* genotype and catheter ablation outcomes in AF patients.

**Methods:** Totally 371 patients who underwent their first catheter ablation for AF were enrolled in this prospective cohort study. They were categorized into four groups based on their *ALDH2* genotypes and habitual alcohol consumption to understand the contribution status to their impact on the risk of AF recurrence. The primary outcome was to determine the proportion of AF recurrence among the four groups during a 1‐year follow‐up period using Kaplan‐Meier analysis. The secondary outcome involved assessing the contributions of each group to AF recurrence and other risk factors using multivariate analysis.

**Results:** This study comprised 239 *ALDH2*‐wild type (147 habitual drinkers) and 132 *ALDH2*‐deficient variant carriers (31 habitual drinkers). Kaplan‐Meier curves indicated that *ALDH2*‐deficient variant carriers with habitual alcohol consumption exhibited the highest proportion of AF recurrence compared with the other groups (p<0.01). In addition, ALDH2‐deficient variant itself was not associated with AF recurrence (hazard ratio [HR]=1.56, p=0.10), *ALDH2*‐deficient variant carriers with habitual alcohol consumption exhibited a higher HR (HR 5.01, p=0.02). Notably, it conferred a higher risk than that for ALDH2 wild‐type patients with habitual alcohol consumption (HR=2.36, p=0.02).

**Conclusions:** While the *ALDH2*‐deficient variant itself showed no correlation with AF recurrence, it emerged as a significant risk factor for AF when accompanied with habitual alcohol consumption. Thus, abstinence from alcohol may be necessary, even after catheter ablation is performed, especially for patients with the *ALDH2*‐deficient variant.

## 8 USING POINT‐OF‐CARE ULTRASOUND TO DETERMINE INCIDENCE AND PREDICTORS OF DEEP VEIN THROMBOSIS AFTER RIGHT SIDED RADIO‐FREQUENCY CATHETER ABLATION

### 8.1 REEMA QAYOOM, HANNAH S. ASGHAR, IRFAN AMJAD LUTFI, FAISAL QADIR, **GHAZALA IRFAN**, AZAM SHAFQUAT

#### 8.1.1 National Institute Of Cardio Vascular Diseases, Karachi, Pakistan

**Introduction:** Femoral venous access is routinely used for radio‐frequency catheter ablation (RFA) procedures. There is limited literature regarding incidence of deep vein thrombosis (DVT), which is often sub‐clinical, following RFCA. Point‐of‐care ultrasound (POCUS) is a cost effective way to diagnose DVT. Identification of DVT risk predictors can direct change in practice to reduce DVT and lay ground for cost effective screening strategies post procedures. The Purpose of this study is to determine the incidence and predictors of DVT after right sided radio frequency cardiac catheter ablation using POCUS.

**Methods:** We conducted a single‐center prospective cross‐sectional study in patients undergoing RFCA for atrioventricular nodal re‐entrant tachycardia and atrioventricular re‐entrant tachycardia with right sided accessory pathway. Within 24 hours post procedure, the participants underwent compression venous duplex ultrasonography using point‐of‐care ultrasound at the common femoral vein and popliteal vein to look for evidence of DVT in cannulated limb. The contralateral limb that was not cannulated was scanned as a control.

**Results:** Total 194 patients were scanned post right sided RFCA procedures. Average age was 43.5+13.2 years and 131 (67.5%) were women. 148(76.3%) underwent AVNRT ablation. 10 (5.2%) patients developed DVT, of which 9 had sub‐clinical DVT. Advancing age (>50 years), greater sum of sheaths used (>3), bed rest (6.2 hours vs 4.7 hours, p=0.179) and longer duration of application of compression dressing (14.9 hours vs 11.3 hours, p=0.051) were identified as risk factors.

**Conclusions:** DVT is not uncommon after right sided catheter ablation and can be diagnosed using POCUS. Most of the DVTs are sub‐clinical; advancing age, greater sum of sheaths used, longer maintenance of post‐procedural bed rest and longer duration of application of compression dressing were identified as risk factors. Routine scanning for DVT after right sided catheter ablation as well as reducing number of sheaths and bed rest should be considered.

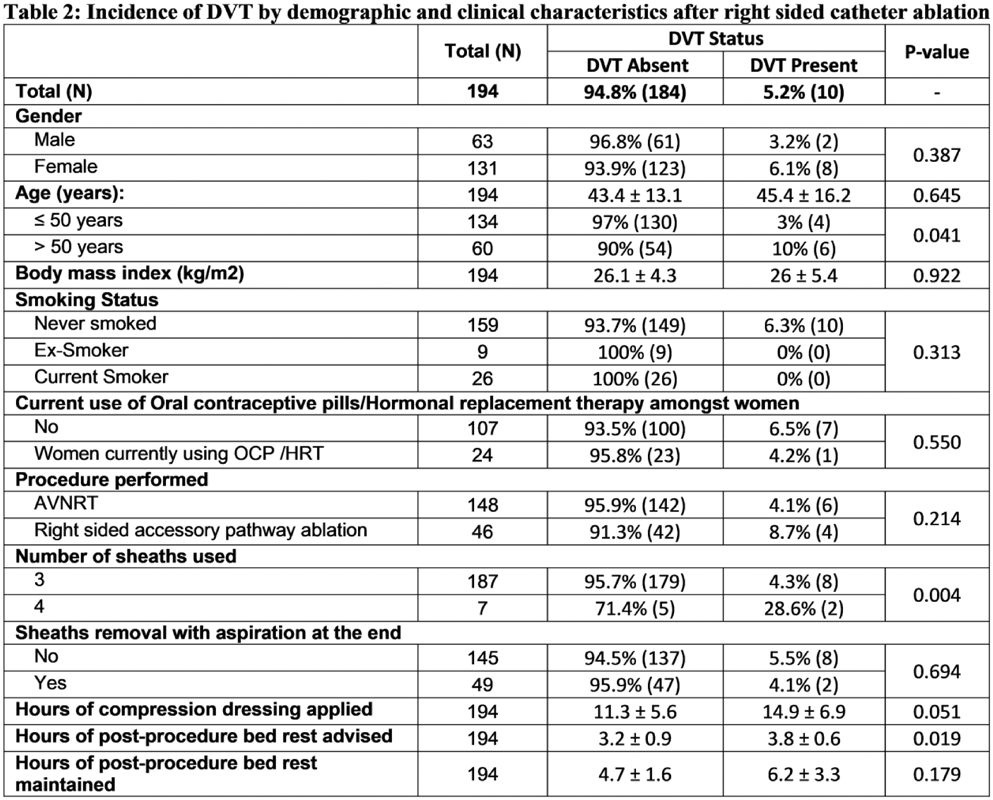

## 9 REVERSIBLE INHIBITION ON SUPERFICIAL MYOCARDIAL EXCITATION BY COLD SALINE APPLICATION THROUGH AN IRRIGATION CATHETER; MAPPING FOR AN IDEAL ABLATION SITE

### 9.1 **TAKUMI KASAI** ^1^, OSAMU SAITOH^2^, AYAKA OIKAWA^2^, HIROKO KOBAYASHI^1^, YASUHIRO IKAMI^3^, YUKI HASEGAWA^3^, SOU OTSUKI^3^, HIROSHI FURUSHIMA^4^, TAKAYUKI INOMATA^3^, MASAOMI CHINUSHI^2^

#### 9.1.1 ^1^Niigata University Graduate School of Health Sciences, Niigata, Japan,^2^Niigata University School of Health Sciences Faculty of Medicine, Niigata, Japan,^3^Department of Cardiovascular Medicine, Niigata University Graduate School of Medical and Dental Sciences, Niigata, Japan,^4^Furushima Clinic, Niigata, Japan

**Introduction:** High flow cold saline application through an irrigation catheter could induce reversible inhibition of the surface myocardial excitation, which may be used to identify an ideal site for radiofrequency (RF) energy delivery, similar to ice‐mapping of cryo‐catheter‐ablation. Experimental studies were performed to examine the distribution of excitation‐inhibited myocardium from the surface during cold saline application.

**Methods:** An open irrigation catheter was positioned either perpendicularly or parallel, with 10‐g contact on coronary perfusing porcine hearts and the contacting myocardium was cooled by high flow cold saline irrigation at 4°C (20 mL/min). Intramyocardial temperature (I‐temp) was measured 2 mm under the myocardial surface. Pacing threshold through the ventricle was measured using eight‐pole needle electrodes (1 mm electrode distance) vertically inserted alongside the ablation catheter, and the 8th electrode of the needle was positioned just under the endocardial surface.

**Results:** By the cold saline application in perpendicular catheter contact, 10 V pacing from the 8th electrode failed to capture the myocardium in 7/10 experiments and I‐temp decreased to 24.8±4.0°C. In parallel catheter contact, these were 9/10 experiments and 24.4±2.6°C, respectively. During the cooling, pacing threshold within 2.5±1.5 mm from the surface increased more than 150% than that before cooling in perpendicular catheter contact, compared to within 2.5±0.8 mm in parallel catheter contact. In both catheter‐contact positions, these increased pacing thresholds during the cold saline application returned to the basal values, and constant myocardial capture by 8th electrode resumed.

**Conclusions:** Regardless of catheter contact position, high flow 4°C cold saline application through an irrigation catheter transiently inhibited myocardial excitation around 2.5 mm under the myocardial surface. This approach may be useful to more safely perform RF energy delivery to sensitive areas of the heart (around AV nodal area, etc.).

## 10 OPTIMIZING LEADLESS ATRIAL PACEMAKER PERFORMANCE THROUGH STRATEGIC REPROGRAMMING

### 10.1 **AMY KLEINHANS**, FANTASIA HUDSON, RAHUL DOSHI

#### 10.1.1 HonorHealth, Scottsdale, AZ

**Introduction:** The recent approval of dual chamber leadless pacemakers marks a significant advancement in cardiac pacing technology. While the safety and efficacy of implantation strategies are well‐documented, long‐term management and performance, particularly concerning battery longevity, remain under observation. Real‐world data has shown shorter atrial leadless pacemaker (aLP) battery life than its ventricular counterpart.

**Methods:** We identified six optimization areas: 1) voltage, 2) pulse width, 3) i2i communication, 4) lower rate limit, 5) mode switch events, and 6) mode changes. A step‐by‐step algorithm was developed for device clinic staff to reprogram patients' devices, aiming to enhance battery life while maintaining aLP efficacy.

**Results:** Twenty patients underwent algorithm‐driven reprogramming, with an initial average battery life of 4.1 years. Post‐reprogramming, the average battery life increased by 1.41 years, a 34.4% improvement (see Figure 1). Significant battery life extension was achieved through optimizations in voltage, i2i communication, lower rate limit, and pulse width. Minimizing inappropriate mode switching also showed potential benefits, though quantifying its impact proved challenging.

**Conclusions:** Dual chamber leadless pacemakers require refined programming strategies beyond traditional techniques. Implementing a structured algorithm enabled clinic staff to fine‐tune settings tailored to each patient, resulting in a substantial 34.4% increase in battery life. These findings underscore the importance of personalized device management to optimize performance and extend battery longevity in leadless pacemakers.

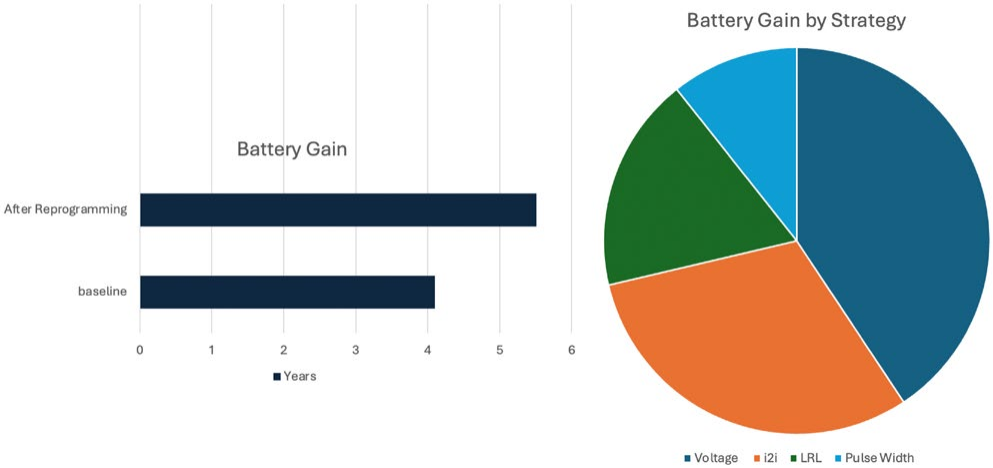

## 11 ADVANCEMENTS IN BATTERY LONGEVITY OF CARDIAC IMPLANTABLE ELECTRONIC DEVICES FROM THE REAL‐WORLD DATA:BATTERY STUDY

### 11.1 **MAIKO KURODA** ^1^, MICHIO NAGASHIMA^1^, MASATAKA NARITA^2^, WATARU SASAKI^2^, NAOMICHI TANAKA^2^, HITOSHI MORI^2^, KAZUHISA MATSUMOTO^2^, TSUKASA NAGANUMA^2^, KOMEI ONUKI^1^, HIROYUKI KONO^1^, TOMONORI KATSUKI^1^, REI KUJI^1^, KENGO KORAI^1^, MASATO FUKUNAGA^1^, KENICHI HIROSHIMA^1^, YOSHIFUMI IKEDA^2^, KENJI ANDO^1^, RITSUSHI KATO^2^

#### 11.1.1 ^1^Kokura Memorial Hospital, Kitakyusyu, Japan,^2^Saitama Medical University International Medical Center, Hidaka, Japan

**Introduction:** Cardiac implantable electronic devices (CIEDs) with longer battery longevity are beneficial as they reduce the patient stress during device exchanges and the risk of infection associated with these procedures. In recent years, several technological improvements have been made in battery structure to enhance device longevity. However, there are no reports on the extent of improvement in battery longevity in the real world.

**Methods:** Patients who underwent CIED exchanges from February 2006 to June 2023 were included in this study. We defined actual battery longevity as the period from the implantation date of the previous device to the date of battery replacement. We also investigated the predicted battery longevity at the time of device implantation, based on manufacturer reports. All patients were divided into five groups according to their initial implantation dates. To minimize follow‐up bias, we excluded the first and last groups and compared data among the middle three groups.

**Results:** A total of 3126 cases (Pacemaker [PM], 2138; Implantable cardioverter defibrillator [ICD], 477; Cardiac resynchronization therapy pacemaker [CRTP], 124; Cardiac resynchronization therapy defibrillator [CRTD], 387) were enrolled. Previous device implantations were performed between November 1993 and December 2020. The predicted battery longevity showed an increasing trend across all device types. However, there was a discrepancy between predicted and actual battery longevity (left panel). The actual battery longevity of PMs, ICDs, and CRTDs showed an extension from Q1 to Q3 (PM 9.7±1.5 months, ICD 11±2.5 months, CRTD 16±2.6 months, p < 0.01), but CRTP devices did not show a significant extension from Q1 to Q3 (CRTP 0.48±6.5 months, p=1.0) (right panel).

**Conclusions:** The real‐world data shows that over the past nearly twenty years, battery longevity has improved by only about one year, indicating that further improvements are needed. Moreover, a discrepancy between predicted and actual battery longevity suggests that a reevaluation of the methods for calculating predicted battery longevity may be necessary.

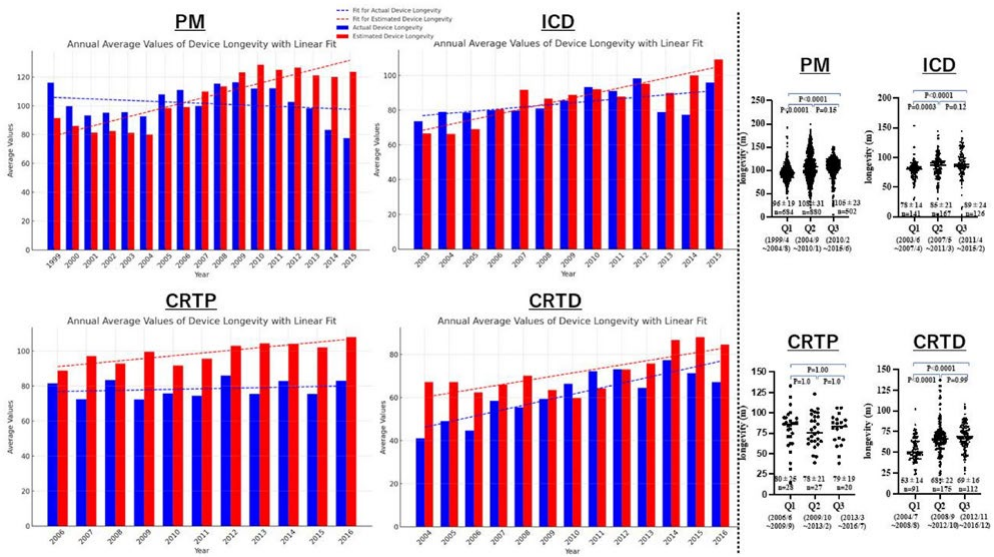

## 12 DETERMINANTS OF TRANSMURAL LESION FORMATION IN PULMONARY VEIN ISOLATION USING THE POLARX CRYOBALLOON AND ULTRA‐HIGH DENSITY MAPPING SYSTEM

### 12.1 **LO CHIEH LING** ^1,2^, TING‐YUNG CHANG^1,2,3^, CHIN‐YUN LIN^1,2^, LI‐WEI LO^1,2^, YENN‐JIANG LIN^1,2^, SHIH‐LIN CHANG^1,2^, YU‐FENG HU^1,2^, FA‐PO CHUNG^1,2^, TA‐CHUAN TUAN^1,2^, TZE‐FAN CHAO^1,2^, JO‐NAN LIAO^1,2^, LING KUO^1,2^, CHIH‐MIN LIU^1,2^, SHIN‐HUEI LIU^1,2^, CHENG‐I WU^1,2^, YU‐SHAN HUANG^1,2^, SHIH‐ANN CHEN^4,2,5^

#### 12.1.1 ^1^Taipei Veterans General Hospital, Taipei City, Taiwan,^2^National Yang Ming Chiao Tung University, Taipei City, Taiwan,^3^National Taipei University of Nursing and Health Sciences, Taipei City, Taiwan,^4^Taichung Veterans General Hospital, Taichung City, Taiwan,^5^National Chung Hsing University, Taichung City, Taiwan

**Introduction:** The POLARx cryoballoon(CB) demonstrated efficacy and safety in the ANTARCTICA study but differs in cryoablation metrics from the Arctic Front Advance. Identifying predictors of transmural lesions post‐POLARx CB ablation remains unexplored. Prior research indicates that complete S wave loss in local unipolar atrial electrograms predicts transmural lesions.

**Methods:** Forty‐three patients with paroxysmal atrial fibrillation were enrolled at Taipei Veterans General Hospital for cryoablation using the POLARx system. Post‐ablation mapping was conducted with the INTELLAMAP ORION™ catheter in all cases.

**Results:** Baseline characteristics are presented in Table 1. Average procedural time was 150.7 ± 43.4 minutes. Pulmonary vein isolation was achieved in all cases, with 3 (7.0%) patients has transient phrenic nerve injury during the procedure. In 62.8% of patients (27 out of 43), complete S wave elimination in all pulmonary veins was observed. Table 2 displays the S wave elimination rate per pulmonary vein alongside procedural parameters. Thawing Time to 0°C (TTT) predicted S wave loss in the right inferior pulmonary vein (RIPV) (P=0.013), with an area under the curve (AUC) of 0.861. A TTT under 16.5 seconds had 90.5% sensitivity and 83.3% specificity. Though not statistically significant, longer TTT showed a trend in predicting S wave loss in the right superior pulmonary vein (RSPV) (P=0.150). Similarly, a shorter Time to Pulmonary Vein Isolation (TTI) trended towards predicting S wave loss in RSPV and RIPV (P=0.516, 0.171, respectively). After cryoablation, the left superior and inferior pulmonary veins (LSPV, LIPV) almost entirely exhibited S wave elimination. The recurrence‐free survival rate over an average follow‐up of 79.7 days (including a 90‐day blanking period) was 4.7%. All patients with recurrence retained residual S wave during the initial procedure.

**Conclusions:** In cryoablation with the POLARx system, TTT may serve as an indicator of transmural lesion formation. The presence of residual S wave could be linked to future recurrence post‐procedure.

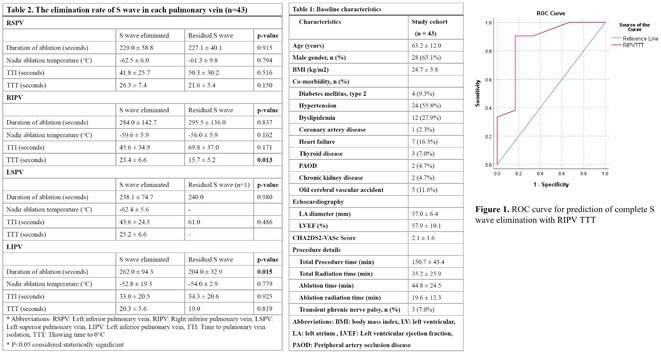

## 13 CHARACTERISTICS OF ADENOSINE SENSITIVE ACCESSORY PATHWAYS IN PATIENTS WITH ATRIOVENTRICULAR REENTRANT TACHYCARDIA

### 13.1 **TAISUKE NABESHIMA**, HITOSHI MORI, KOTA NAGAOKA, RITSUSHI KATO, NAOKATA SUMITOMO

#### 13.1.1 Saitama Medical University International Medical Center, Hidaka, Japan

**Introduction:** Conduction block in accessory pathways (AP) by adenosine triphosphate (ATP) is relatively rare phenomenon and often considered to be a feature of APs with decremental property as observed in cases of PJRT. However, the whole clinical picture of ATP sensitive APs has not been elucidated.

**Methods:** From 2019/1 to 2023/9, 131 consecutive patients with atrioventricular reentrant tachycardia who underwent a 1^st^ catheter ablation using 3D mapping in our institute were enrolled in this study. The patients are divided into 77 cases < 16‐years‐old (pediatric group), and the other 54 cases (adult group). The anatomical location and electrophysiological characteristics of overall APs and adenosine sensitive APs were investigated respectively in each group.

**Results:** Table 1 shows each group's background. For the age at the time of ablation, the 12‐14 age group was dominant (31%) in the pediatric group, but almost the same for each age group in the adult group. Regarding the AP locations, types A, B, C were 50%, 38%, and 4% respectively in the pediatric group and 80%, 9%, and 9% in the adult group. The retrograde AP effective refractory period did not significantly different among the groups. According to the adenosine sensitive APs, 6 cases (4.5%) were identified and 5 cases out of them belonged to the pediatric group (Table 2). All the adenosine sensitive APs were located in left atrium and possessed only retrograde conduction. 3 cases (50%) had decremental property. Median ERP of adenosine sensitive APs were 400ms, longer than that of overall APs(295ms).

**Conclusions:** Adenosine sensitive APs were found in younger patients and more often located in left atrium, and not limited to PJRT cases. These APs have longer ERP and conduction time.

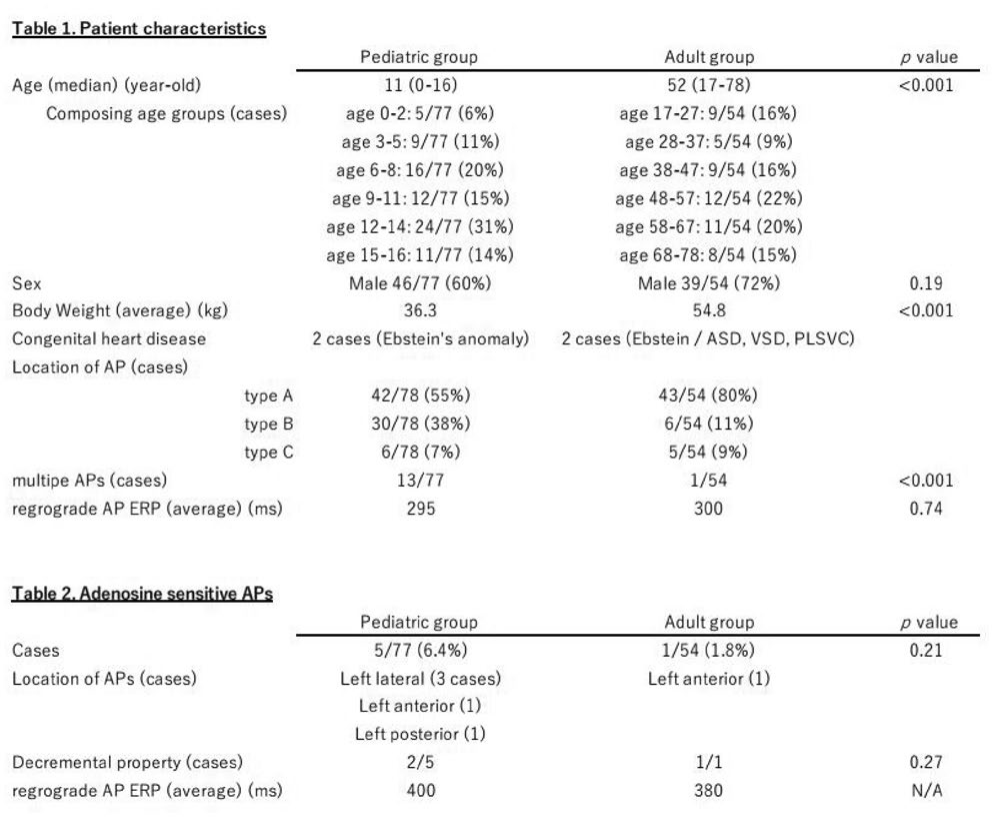

## 14 THE RELATIONSHIP BETWEEN ATRIAL FIBRILLATION RECURRENCE AND LEFT ATRIAL FUNCTION AFTER CATHETER ABLATION IN PATIENTS WITH ATRIAL FUNCTIONAL MITRAL REGURGITATION

### 14.1 **KAZUHIRO NAGAOKA** ^1^, YASUSHI MUKAI^2^, HIROSHI KOJIMA^1^, SHUNSUKE KAWAI^2^, SUNJI HAYASHIDANI^1^, HIDEKI TASHIRO^1^

#### 14.1.1 ^1^St.Mary's Hospital, Kurume, Japan,^2^Japanese Red‐Cross Fukuoka Hospital, Fukuoka, Japan

**Introduction:** We previously reported that catheter ablation (CA) improved atrial functional mitral regurgitation (A‐FMR) via maintaining sinus rhythm. Recently, it was reported that left atrial function predicts atrial arrhythmia recurrence following CA in patients with long‐standing persistent atrial fibrillation. The purpose of this study was to compare the difference in left atrial function before and after CA and their relations to atrial arrhythmia recurrence in patients with A‐FMR.

**Methods:** Thirty‐one patients with A‐FMR undergoing initial CA for AF in two hospitals were investigated. LA structure and function were assessed by 2‐dimentional volume and speckle tracing strain measurements of LA reservoir (LASres), conduit (LAScd), and contractile strain (LASct) before and after CA.

**Results:** Mean age of patients was 74.9±6.9 years, 38.7% were male, and average BNP levels was 241.4±183.9 pg/ml. CA increased left atrial strain (LASres, LAScd, and LASct) and left atrial empting fraction (LAEF), and reduced left atrial volume index with maximum and minimum (LAVI max and LAVI min), associated with a reduction in the degree of mitral regurgitation (Table1). Patients without atrial arrhythmia recurrence had higher LASct and smaller LAVI min after CA under sinus rhythm than those with recurrence (Table2).

**Conclusions:** CA improves left atrial function and reduces mitral regurgitation in patients with A‐FMR. Higher LASct and a smaller LAVImin under sinus rhythm after CA may predict no‐recurrence in patients with A‐FMR.

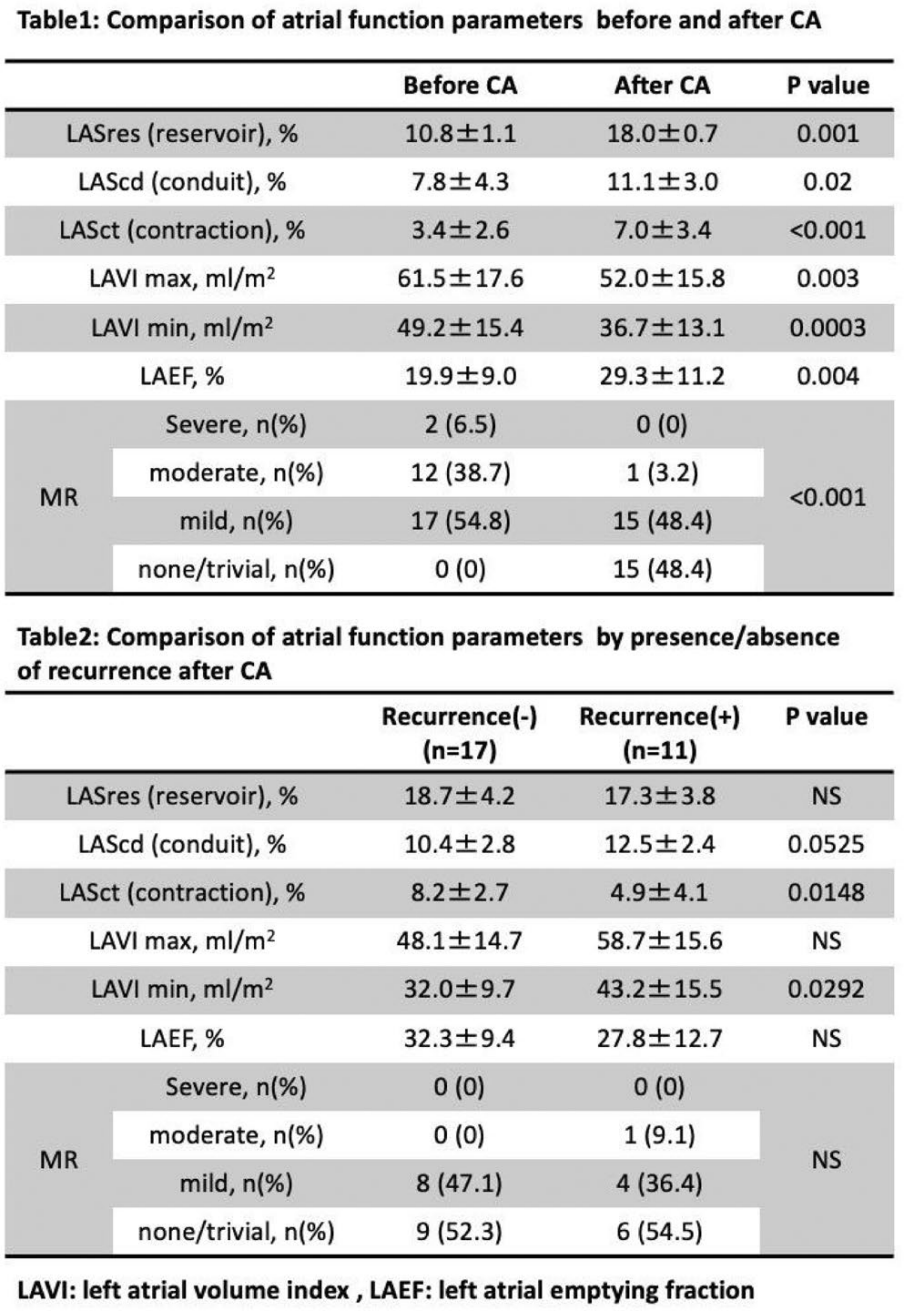

## 15 IMPLANTATION OF STYLET‐DRIVEN LEADS FOR LEFT BUNDLE BRANCH AREA PACING GUIDED BY NEW DELIVERY CATHETERS: A MULTI‐CENTER EXPERIENCE

### 15.1 **DEVI NAIR** ^1^, WENWEN LI^2^, ERIC JOHNSON^2^, KYUNGMOO RYU^2^, YOEL VIVAS^3^, BRANDON DOTY^1^, KIROLLOS GABRAH^1^, ASIM SYED^3^, BRETT ATWATER^4^

#### 15.1.1 ^1^St. Bernards Medical Center & Arrhythmia Research Group, Jonesboro, AR,^2^Abbott, Sylmar, CA,^3^The Arrhythmia Center of South Florida, Boca Raton, FL,^4^Inova Heart and Vascular Institute, Fairfax, VA

**Introduction:** Stylet‐driven leads (SDL) are increasingly used for left bundle branch area pacing (LBBAP). This study aimed to report the clinical experience of LBBAP implant using the only SDLs with FDA approved LBBAP labelling and newly introduced fixed curve delivery catheters.

**Methods:** Implant procedural data were collected from consecutive patients receiving LBBAP using stylet‐driven leads (Tendril™ STS 2088TC and UltiPace™ LPA1231, Abbott, USA) and CPS Locator™ 3D catheter (3 curves: S/M/L, Abbott, USA) between April and December 2023 at three centers. LBBAP implant success and lead location were determined following the latest clinical consensus using left ventricular activation time (LVAT), fluoroscopy, and paced QRS morphology.

**Results:** A total of 80 patients (Age 73±13 years; Male 58%; LVEF 57%±10%; NYHA Class 2.2±0.7; baseline QRS duration 118±33 ms) underwent LBBAP implant (Devices: 90% DR Pacemakers, 7% CRT‐P, and 3% CRT‐D; Leads: 93% Tendril, 7% UltiPace). Primary pacing indications were SSS (67.5%) and AVB (30.0%). Distribution of the delivery catheter curves used was 5%, 88%, and 8%, for small, medium, and large, respectively. Successful LBBAP implant was achieved in 76/80 (95%) patients, with a single attempt (90%) or 2 attempts (10%). Leads were implanted at LBB trunk (22%), LBB fascicles (54%), and LV septum (19%). LVAT was 68±13 ms, V6‐V1 interval was 41±15 ms, and fluoroscopy time was 6.2±4.3 mins. Time from venous access to lead fixation was 19±9 mins. LBBAP capture threshold was 1.0±0.5 V at pulse width of 0.4 ms. Pacing impedance was 630±83 ohms; R‐wave sensing was 9.2±4.1 mV. Paced QRS duration was 118±19 ms, with a V1 morphology of qR (52%), Qr (24%), QR (20%), RSr’ (2%), and QS (2%). There were no LBBAP lead related procedural complications.

**Conclusions:** LBBAP implants using SDLs and the new fixed curve delivery catheters were safe and effective with a high success rate in a multi‐center clinical experience.

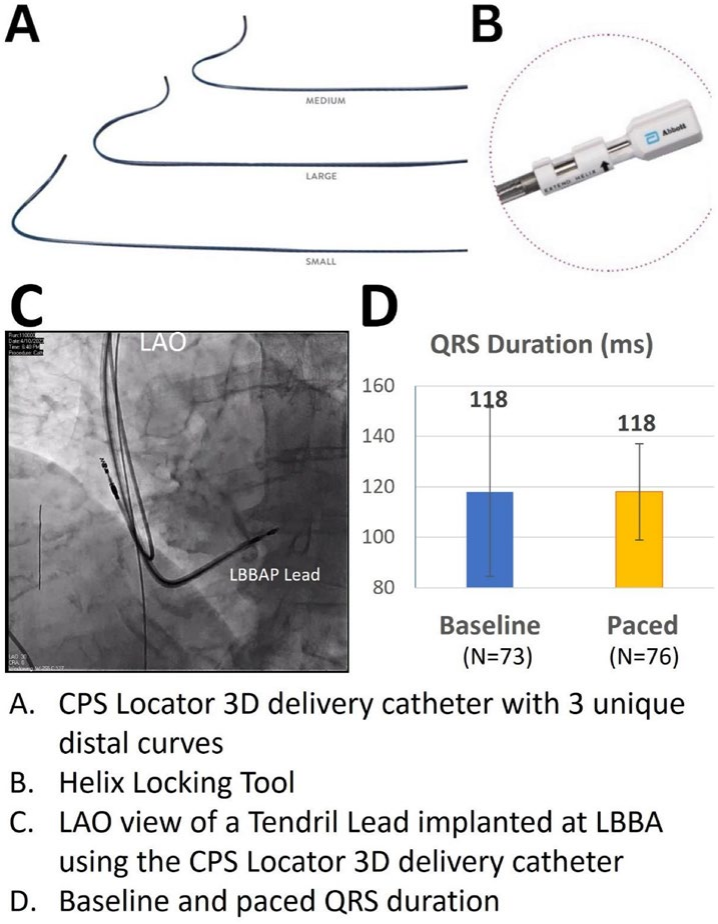

## 16 FRAILTY AND PRE‐FRAILTY IMPROVEMENT AFTER PULMONARY VEIN ISOLATION IN JAPANESE PATIENTS WITH ATRIAL FIBRILLATION

### 16.1 **YUSUKE OKUYAMA** ^1^, TOSHIKI KAZUMA^2^, KOUHEI UEDA^1^, DAISUKE HATTORI^1^, MIYU SUGIMOTO^1^, CHISAKI TAKAHARA^1^, KEIKOKU KASHO^1^, ATSUSHI TAMURA^1^, MASAHIKO JIKAN^1^, KANTA NAKAJIMA^1^, KAZUNORI MASUDA^1^, SHUNZO MATSUOKA^1^, YOSHIHISA NAKAGAWA^3^

#### 16.1.1 ^1^Uji‐Tokushukai Medical Center, Uji, Japan,^2^Nagoya Heart Center, Nagoya, Japan,^3^Shiga University of Medical Science, Otsu, Japan

**Introduction:** The number of patients with atrial fibrillation (AF) is increasing due to the growth in healthy life expectancy in aging society in Japan. Elderly patients with AF who need pulmonary vein isolation (PVI) is also increasing. The symptoms brought by AF may cause decreased activity, depression, reduced participation in social activities, and progression of frailty. This study aimed to evaluate whether PVI has an effect on improving frailty. Effects of PVI on causes of frailty progression were also evaluated.

**Methods:** A total of 200 consecutive patients who underwent PVI in our hospital were included in this study. Frailty of participants was assessed using the Clinical Frailty Score (CFS). Causes of frailty was also assessed by questions used in Japan to evaluate frailty and pre‐frailty in the elderly, including questions on reduced activity, physical function, and depression, before and 3 months after PVI. CFS and causes of frailty were scored and the values before and after PVI were compared. The correlation between the degrees of improvement in these scores and age was also investigated.

**Results:** CFS significantly improved after PVI (2.0±1.0 vs 1.5#177;0.7, p<0.01). Scores for frailty and pre‐frailty also significantly improved after PVI, especially in reduced activity (1.3#177;1.5 vs 0.4#177;1.1, p<0.01). There was a positive correlation between the decrease in CFS and age, with sensitivity of 0.67 and specificity of 0.79 for the ROC curve when the cutoff age was 74.5 (AUC=0.81). In patients aged 75 years and older, CFS, scores for reduced activity, physical function, and depression significantly decreased after PVI (2.5#177;0.9 vs 1.8#177;0.7, p<0.01, 1.9#177;1.6 vs 0.6#177;1.2, p<0.01, 0.9#177;1.1 vs 0.4#177;0.8, p<0.01, and 0.8#177;0.9 vs 0.1#177;0.5, p<0.01, respectively). In addition, scores for reduced activity and depression improved to the same level as patients under 75 years.

**Conclusions:** Frailty were improved and some causes of frailty progression was reduced by PVI. Furthermore, the effect was more marked in patients over 75 years.

## 17 RECOMMENDATIONS FOR ATRIAL FIBRILLATION SCREENING IN THOSE WITH RHEUMATIC HEART DISEASE: A POLICY CONTENT ANALYSIS

### 17.1 **JESSICA ORCHARD** ^1^, DIANA FOO^2^, PRIYANSH SHAH^3^, JULIAN HÖVELMANN^4^, MENGLU OUYANG^5^, JAMEEL AHMAD^6^, CAIO AM TAVARES^7^, ELIZABETH PARATZ^8^

#### 17.1.1 ^1^The University of Sydney, Sydney, Australia,^2^Clinical Research Centre Sarawak General Hospital, Kuching, Malaysia,^3^Jacobi Hospital/Albert Einstein College of Medicine, New York, NY,^4^Cape Heart Institute, Faculty of Health Sciences, University of Cape Town, Cape Town, South Africa,^5^The George Institute for Global Health, Sydney, Australia,^6^Kano Independent Research Centre Trust/ Bayero University Kano, Kano, Nigeria,^7^Hospital Israelita Albert Einstein, São Paulo, Brazil,^8^St Vincent's Institute of Medical Research, Melbourne, Australia

**Introduction:** Rheumatic heart disease (RHD) is a leading global health problem, with a prevalence of 513/100,000 people worldwide. RHD heavily impacts low‐ and middle‐income countries (LMICs). Atrial fibrillation (AF) is a major complication of RHD. Almost one‐third of RHD patients have AF, incidence rises steeply after diagnosis and stroke is a major cause of RHD morbidity and mortality. Despite this, AF screening in RHD patients is not widely recommended or performed.

**Methods:** We conducted a narrative policy review and content analysis to examine AF screening recommendations for RHD patients. Relevant policy documents (guidelines, policies, practice guidance and consensus statements on AF and/or RHD or valvular heart disease) from around the world were identified. For guidelines issued more than once, we used the most recent version only. Content analysis was used to describe these recommendations qualitatively.

**Results:** In total, 19 relevant policy documents were reviewed (10 for RHD or valvular heart disease and 9 for AF). The only document that included specific recommendations for AF screening in those with RHD was the Asia Pacific Heart Rhythm Society (APHRS) 2021 practice guidance on AF screening. The APHRS guidance document recommended AF screening for RHD patients with high‐risk features (age >50 years, left atrial dimension >4cm, mitral valve area <1cm^2^, mitral valve calcification, mitral valve gradient >10 mmHg or New York Heart Association Class II or above). Noting the heterogeneity in Asia‐Pacific countries, recommendations were divided into levels that are applicable to different countries.

**Conclusions:** Although AF is a common complication of RHD, only one policy document was identified that provided specific recommendations for AF screening in patients with RHD. Recent work on RHD has shown that RHD is not adequately prioritised as a global priority for several reasons, including the perception that it mainly affects LMICs, and highlighted the need for ongoing advocacy. Given the global prevalence of RHD, further work is needed to inform and develop appropriate AF screening guidelines tailored to different settings.

## 18 PROGNOSTIC IMPACT OF DIAGNOSIS‐TO‐ABLATION TIME ON OUTCOMES FOLLOWING CATHETER ABLATION IN AF AND LEFT VENTRICULAR SYSTOLIC DYSFUNCTION

### 18.1 **LOUISE SEGAN** ^1,2^, PETER KISTLER^1,2^, DAVID CHIENG^1,2^, ROSE CROWLEY^1,2^, JEREMY WILLIAM^1,2^, KENNETH CHO^1,2^, HARIHARAN SUGUMAR^1^, LIANG‐HAN LING^1^, ALEKSANDR VOSKOBOINIK^1^, JOSHUA HAWSON^3^, JOSEPH MORTON^3^, GEOFF LEE^3^, PRASHANTHAN SANDERS^4^, JONATHAN KALMAN^3^, SANDEEP PRABHU^1,2^

#### 18.1.1 ^1^Alfred Health, Melbourne, Australia,^2^Baker Heart and Diabetes Institute, Melbourne, Australia,^3^Royal Melbourne Hospital, Melbourne, Australia,^4^Royal Melbourne Hospital, Adelaide, Australia

**Introduction:** The optimal timing of catheter ablation in individuals with atrial fibrillation(AF) and left ventricular systolic dysfunction(LVSD) remains uncertain. We sought to examine whether AF diagnosis to ablation time(DAT) influences outcomes following catheter ablation in AF with left ventricular systolic dysfunction.

**Methods:** We evaluated individuals with AF and LVSD (LVEF<50%) referred for catheter ablation from the CAMERA‐MRI and CAPLA randomized trials. We compared AF recurrence, AF burden, change in left ventricular systolic function and left ventricular recovery at 12 months in DAT </>1 year. We also dichotomised DAT according to the median (24 months) and compared outcomes in DAT </>24 months.

**Results:** 210 individuals with AF and LVSD with median DAT of 24 months were identified. AF and LVSD were co‐diagnosed in 24.3%. Individuals with shorter DAT(<1yr) had lower baseline LVEF (34.5±9.2% vs 37.5±9.1% DAT>1 year,p=0.025), with a lower burden of global and posterior LA wall scar (<0.05mV; both p<0.05).At 12 months, 69.4% with shorter DAT (<1year) were free from recurrent atrial arrhythmias off antiarrhythmic drug therapy vs 53.6% with longer DAT (HR 1.63, 95% CI 1.01‐2.65 LogRank p=0.040, figure 1a). Median AF burden was 0% in both groups (shorter DAT: IQR 0.0‐2.0% vs longer DAT: IQR 0.0‐7.3%,p=0.017, figure 1b).Shorter DAT was associated with higher LVEF at 12 months (55.3% vs 51.0%, p=0.009) and greater LVEF improvement (+20.8±13.0% vs +13.9±13.2% in DAT >1 year, p<0.001). LV recovery(LVEF>50%) was greater with shorter DAT (54 (75.0%) vs longer DAT (57.2%, p=0.011). Shorter DAT was associated with lower rates of unplanned hospitalisation and direct cardioversion(both p<0.05).

**Conclusions:** In individuals with AF and LVSD, shorter DAT was associated with greater arrhythmia‐free survival, lower AF burden, greater left ventricular recovery and lower rates of hospitalisation and DCR procedures at 12 months, highlighting the prognostic benefit of early catheter ablation in AF and LVSD.

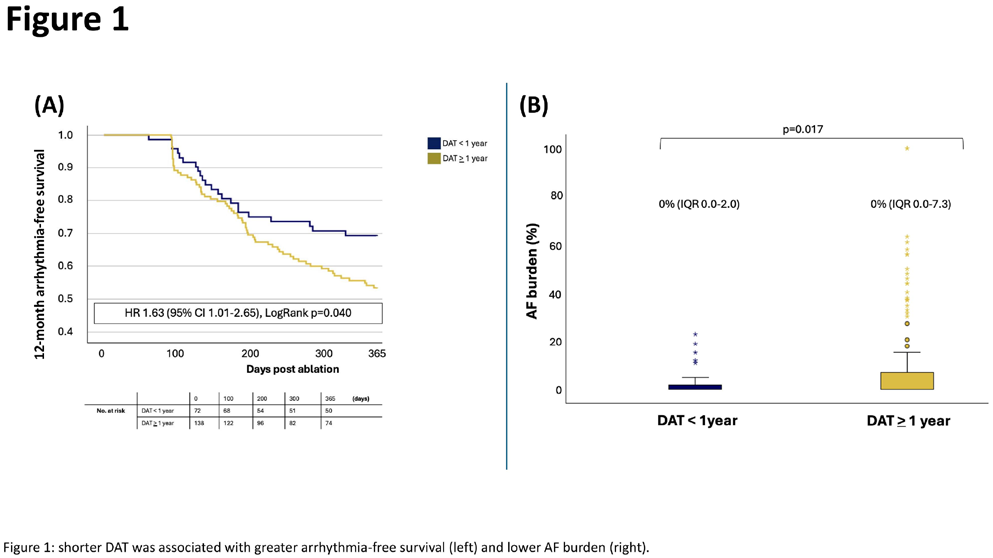

## 19 COMPARISON OF CLINICAL FEATURES OF CARDIAC AMYLOIDOSIS IN PATIENTS WITH CLINICALLY DIAGNOSED VS. DETECTED BY ATRIAL BIOPSY DURING ATRIAL FIBRILLATION ABLATION

### 19.1 **KODAI SHINZATO**, TAKANORI YAMAGUCHI, TOYOKAZU OSYUBO, KOICHI NODE

#### 19.1.1 Saga university, Saga, Japan

**Introduction:** Background: The association between cardiac amyloidosis and atrial fibrillation (AF) is well known. However, the clinical characteristics of patients with AF in whom amyloid deposition is identified in the atria during AF ablation are unknown. Objective: To compare the clinical features of patients who were clinically diagnosed with cardiac amyloidosis with those detected by atrial biopsies during AF ablation.

**Methods:** Methods: A total of 58 patients who were clinically diagnosed with cardiac amyloidosis (CA) based on right ventricular biopsy in our institutes between 2002 and 2022 excluding CA diagnosed by other modalities were examined (clinical CA group). In a prospective observational cohort study based on histological assessment of atrial tissue by right atrial biopsy during AF ablation between 2020 and 2024, 41 patients (7.1%) had amyloid deposition among 577 patients enrolled (atrial biopsy‐detected CA group). Amyloid deposition was assessed by Congo red staining and apple‐green birefringence. Then amyloid histological typing was performed using immunohistochemistry.

**Results:** Results: Compared to the clinical CA group, the atrial biopsy‐detected CA group were older, however, had lower cardiac troponin T level, higher ejection fraction, less left ventricular diastolic dysfunction, and less left ventricular hypertrophy, suggesting that CA was less advanced in the atrial biopsy‐detected CA group. Comparisons of clinical characteristics are shown in the table. There was no difference in the frequency of ATTR and AL type between the two groups, however in the atrial biopsy‐detected CA group, ANP type was identified in three cases.

**Conclusions:** Conclusions: Atrial biopsy revealed amyloid deposition in the atrium in 7% of patients undergoing AF ablation. These patients represent an earlier stage of CA than those with clinically diagnosed CA. Atrial biopsy during AF ablation would be useful for the early detection and diagnosis of CA.

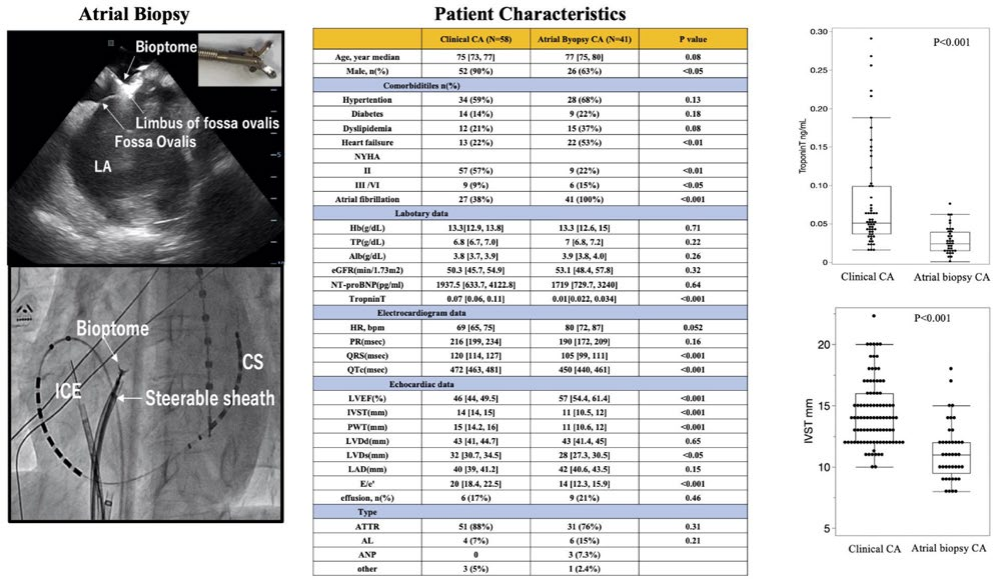

## 20 EFFICACY AND SAFETY RESULTS OF A NOVEL SMALL‐DIAMETER DEFIBRILLATION LEAD BY BODY HABITUS: LEADR TRIAL ANALYSIS

### 20.1 **MORIO SHODA** ^1^, PAMELA MASON^2^, PRASHANTHAN SANDERS^3^, BERT HANSKY ^4^, PAOLO DE FILIPPO^5^, MAULLY SHAH^6^, SURINDER KHELAE^7^, HUNG‐FAT TSE^8^, XINGBIN LIU^9^, PIPIN KOJODJOJO^10^, ANDY ST. MARTIN^11^, GEORGE CROSSLEY^12^

#### 20.1.1 ^1^Tokyo Women's Medical University Hospital, Tokyo, Japan,^2^University of Virginia Medical Center, Charlottesville, VA,^3^Royal Adelaide Hospital, Tokyo, Australia,^4^Städtische Kliniken Bielefeld gem. GmbH ‐ Klinikum Mitte , Bielefeld, Germany,^5^ASST Papa Giovanni XXIII, Bergamo, Italy,^6^The Children's Hospital of Philadelphia , Philadelphia, PA,^7^Institut Jantung Negara, Kuala Lumpur, Malaysia,^8^The University of Hong Kong, Pok Fu Lam, Hong Kong,^9^West China Hospital Sichuan University, Sichuan, China,^10^Asian Heart and Vascular Centre, Singapore, Singapore,^11^Medtronic, Inc., Mounds View, MN,^12^Vanderbilt University Medical Center, Nashville, TN

**Introduction:** With ICD/CRT‐D patients living longer, interest in reliable leads with targeted placement is increasing. The Lead EvaluAtion for Defibrillation and Reliability (LEADR) trial assessed the novel, small‐diameter, lumenless, catheter‐delivered, OmniaSecure defibrillation lead, based on the established SelectSecure 3830 pacing lead platform.

**Methods:** The worldwide LEADR Pivotal trial enrolled de novo ICD/CRT‐D indicated patients. The primary efficacy endpoint was successful defibrillation at implant. The primary safety endpoint was freedom from study lead‐related major complications at 6 months. For the primary endpoints, patients were stratified by tertiles for body mass index (BMI), height, and weight, and defibrillation efficacy and safety were evaluated within these subgroups.

**Results:** In total, 675 participants were enrolled, of which 657 had an implant attempt. The defibrillation success rate for all inducible patients in the study was 97.5% (116/119; in a pre‐specified subgroup, the lower bound of the 95% credible interval was 91%, exceeding the performance goal of 88%) and no statistical difference was observed in success between patients by tertiles of BMI (p=0.375), height (p=0.345), or weight (p=0.645). The Kaplan‐Meier estimate of the rate of freedom from lead‐related major complications at 6‐months in all patients with an implant attempt was 97.1% (the lower bound of the 95% credible interval was 95.2%, exceeding the performance goal of 90%), and no statistical difference was observed in the freedom from major complications by tertiles of BMI (p=0.481), height (p=0.957), or weight (p=0.964). There were zero fractures observed through 12.7 ± 4.8‐month follow‐up.

**Conclusions:** The results from the LEADR trial demonstrate that the OmniaSecure lead exceeded prespecified primary endpoint performance goals for safety and efficacy, demonstrating high defibrillation success and a low occurrence of lead‐related major complications with zero lead fractures. There were no significant differences observed by BMI, height or weight, indicating consistent performance of the OmniaSecure lead across body habitus.

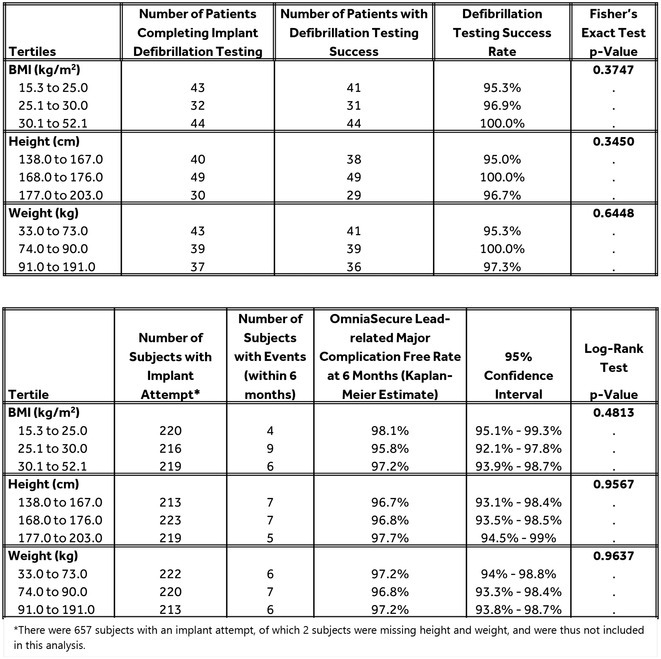

## 21 EVALUATING LESION INTEGRITY WITH DIAMOND TEMP SYSTEM

### 21.1 **USMAN SIDDIQUI** ^1^, AQEEL KHANANI^2^

#### 21.1.1 ^1^Advent Health Orlando, Orlando, FL,^2^Lakeland Regional Health, Lakeland, FL

**Introduction:** Pulmonary vein (PV) isolation by temperature‐controlled (TC) radiofrequency ablation (RFA) is regaining popularity due to tissue‐temperature (TT) feedback from thermocouples attached at the distal tip. The Diamond Temp (DT) ablation catheter system is uniquely able to monitor real time tissue temp during RFAs.

The DT catheter tip has a 1.1mm split tip electrode, allowing bipolar pacing during RFA. Loss of capture (LOC) with High output pacing (HOP) has been considered a surrogate to assess lesion transmurality. This prospective study evaluated the utility of DT catheter distal pacing to achieve durable lesions. Standard guidelines for TC ablation recommend achieving target TT of 60°C to produce durable lesions. The purpose of this study was to assess whether LOC is achieved at target TT of 60°C.

**Methods:** While target enrollment is 50 patients, these findings are based on analysis of the first 16 patients, all of whom had symptomatic, drug‐refractory paroxysmal AF.

Point‐by‐point RF lesions were delivered at 4 segments (sup, inf, ant, post) around the PVs. Ablation was performed in a TC mode with max temp setting of 60°C and max power of 50 watts. HOP was performed prior to start of ablation and discontinued once LOC was observed. Time taken to LOC was recorded. Intracardiac echocardiography (ICE) was used to guide contact and lesion formation.

**Results:** Overall mean time to LOC was 3.32 sec. Ant sites exceeded this mean. 92.81% of locations achieved LOC within 2‐5 sec of ablation. The right ant PV had the longest time at 3.72 sec, exceeding the overall mean of 3.32 sec. All patient achieved FPI.

**Conclusions:** Reaching target TT of 60°C alone may not be sufficient to achieve LOC around PVs. Ablation duration of 2‐5 sec led to LOC in 92.81% of lesions. Certain anatomical regions like ant locations required prolonged ablation time. Tissue thickness on ICE correlated directly with time required to LOC. This analysis shows that TC ablation with DT is effective; however, a therapeutic stable temperature of 60°C for at least 4 sec may be needed to achieve durable lesions around PVs. Tissue thickness on ICE and stability of temperature appear to be independent variables affecting duration prior to LOC.

## 22 CHRONIC PERFORMANCE OF A NEW STYLET‐DRIVEN PACEMAKER LEAD IN THE LEFT BUNDLE BRANCH AREA AND RIGHT ATRIUM

### 22.1 MATTHEW BERNABEI^1^, SANDEEP BANSAL^1^, JEFFREY ARKLES^1^, STEVEN STABINGER^2^, **MARK SPROWLS** ^2^, LEYLA SABET^2^, R. WARD PULLIAM^1^

#### 22.1.1 ^1^Lancaster General Health, Lancaster, PA,^2^Abbott, Sylmar, CA

**Introduction:** A new stylet‐driven lead (SDL) was designed to provide optimal torque transfer to facilitate implantation at deep septal and traditional endocardial pacing sites. This is the first report of its electrical performance when used for LBBAP and right atrial (RA) pacing.

**Methods:** Ninety‐four SDLs (UltiPace™ LPA1231; Abbott, USA) were implanted in 66 patients between September and November 2023. Leads were implanted in the LBBA (n=49) and RA (N=45). LBBA implant procedures were performed using the CPS Locator 3D catheter (Abbott, USA) with implant location confirmed using fluoroscopy and 12‐lead ECG. Leads were used with a single‐chamber pacemaker (20% of patients), dual‐chamber pacemaker (62%), dual‐chamber ICD (5%), CRT‐P (8%), or CRT‐D (5%). Implant and 3‐month follow‐up data was collected, with pacing capture thresholds (PCTs) measured at 0.4 or 0.5ms pulse width. Implant data was unavailable for 5 patients; 2 additional patients were lost to follow‐up.

**Results:** For leads implanted at the LBBA (N=49), PCT, sensing amplitude, and pacing impedance were 0.8 ± 0.3V, 9.3 ± 2.9mV, and 634 ± 110Ω at implant and 0.7 ± 0.2V, 12.5 ± 5.3mV, and 482 ± 81Ω at 3‐month follow‐up. For leads implanted in the RA (N=45), PCT, sensing amplitude, and pacing impedance were 1.0 ± 0.4V, 2.5 ± 1.5mV, and 488 ± 69Ω at implant and 0.7 ± 0.2V, 3.0 ± 1.5mV, and 458 ± 58Ω at 3‐month follow‐up. There was no observed helix or lead malfunction through 3 months of follow‐up. Electrical performance improved from implant to 3‐month follow‐up, with a 13% and 30% decrease in PCT in addition to a 30% and 20% increase in sensing amplitude for leads implanted in the LBBA and RA respectively. LBBA and RA PCTs were ≤1.25V at 3‐month follow‐up for all patients. Electrical performance was comparable to commercially available leads with equivalent intended use.

**Conclusions:** The new SDL, with FDA approved indications for use for both LBBAP and traditional endocardial pacing, demonstrated adequate electrical performance through 3 months of follow‐up with marked improvements in PCT and sensing post‐implant.

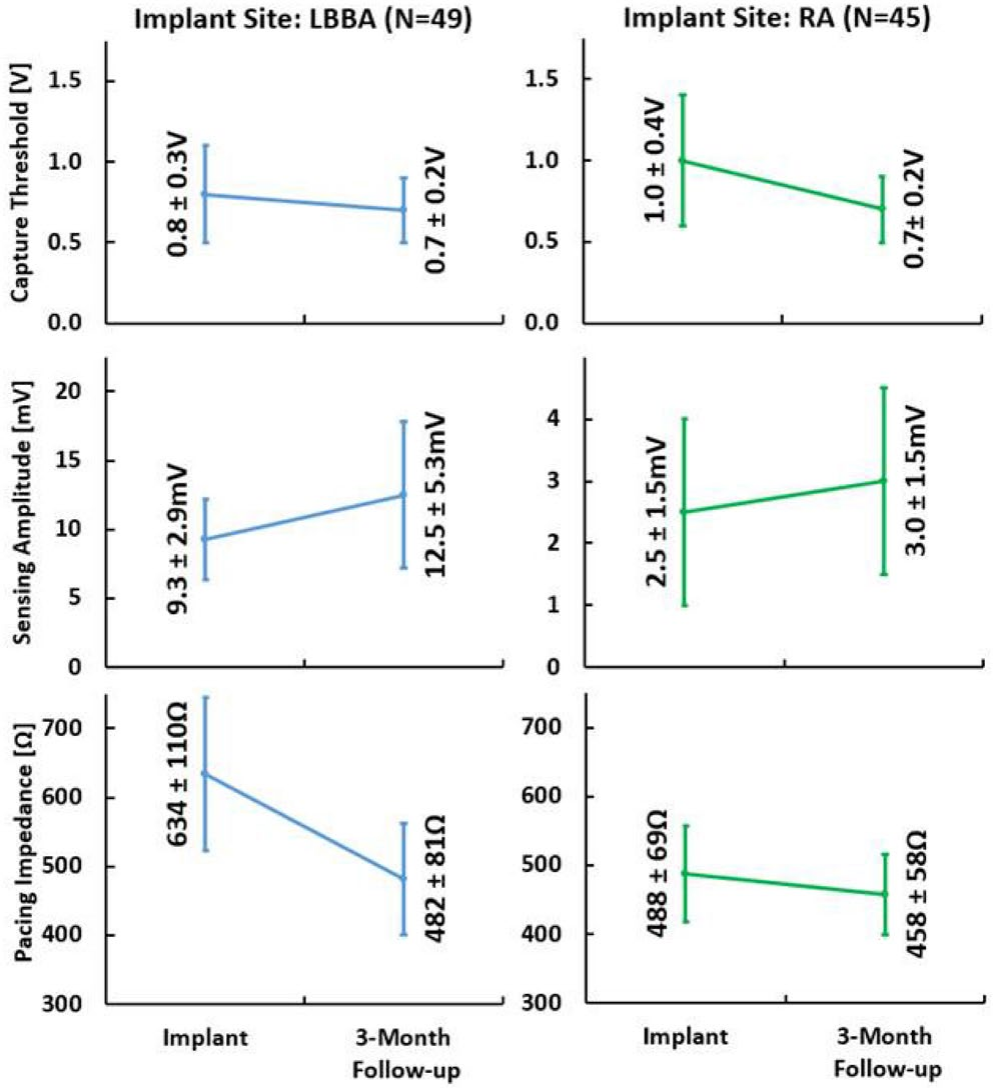

## 23 INCIDENCE OF PERICARDIAL EFFUSIONS IN THE IMPLANTATION OF TEMPORARY PERMANENT PACEMAKERS VS TEMPORARY PACING WIRES

### 23.1 **TORI STAMATOPOULOS**, KATIE NGUYEN, VIET DANG, ASHLEY TOWNEY, TIMMY PHAM, PHOEBE GANIS, KATHARINE BATE, JAMIE CHAM, HUI TIE, TUAN NGUYEN, ROHAN RAJARATNAM, HANY DIMITRI, SIDNEY LO, DOMINIC LEUNG, ADAM LEE

#### 23.1.1 Liverpool Hospital, Sydney, Australia

**Introduction:** Temporary pacing wires (TPWs) are commonly used in patients who need urgent pacing for bradycardia either as a bridge to a permanent device or to rhythm recovery. Temporary permanent pacemakers (TPPs) are an alternative means of providing temporary pacing.

**Methods:** A retrospective single centre (Liverpool Hospital, Sydney, Australia) study was conducted in which data on TPWs was obtained during a 2022 audit and compared to TPPs implanted since the service was commenced in Aug 2023. TPW insertion involved delivery of a 6F fixed curve pacing catheter via the femoral or internal jugular veins to the RV apex. TPPs involved delivery of an active fixation pacemaker lead to the RA or RV which was then connected to a resterilised generator.

**Results:** 38 TPWs were inserted in 37 patients. 6 out of 38 (16%) resulted in RV perforation. All perforations occurred in TPWS inserted via the femoral route. 3 of 6 (50%) patients with TPW perforations died within 30 days. All 3 patients died due to hemodynamic instability/cardiac arrest resulting from the perforation. No pericardial effusions occurred in 10 patients who underwent 11x TPP insertions. The singular complication that occurred with the TPP was a lead dislodgement requiring revision.

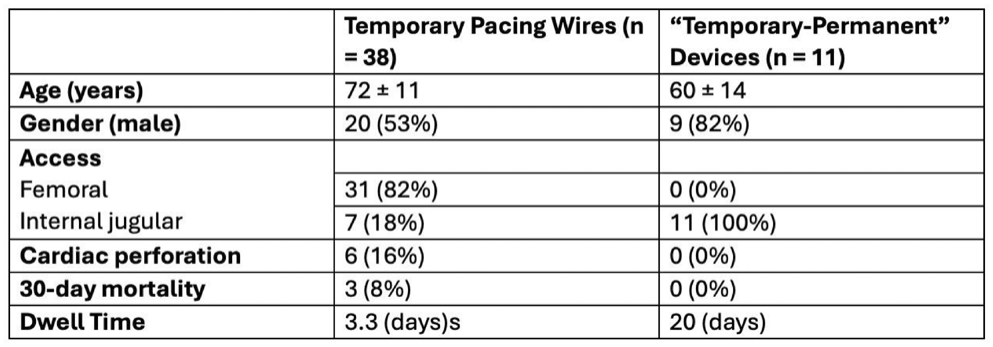

**Conclusions:** TPWs, particularly those inserted via the femoral vein approach, are associated with a high rate of cardiac perforation resulting in tamponade and death. TPP insertion may be a safer method of providing temporary pacing in these patients.

## 24 LEFT BUNDLE BRANCH AREA PACING EFFICACY, FEASIBILITY, AND LONG‐TERM OUTCOMES: A PROSPECTIVE COHORT STUDY

### 24.1 **LUKAH Q. TUAN** ^1,2,3^, JENISH SHROFF^1,2,3^, NATASHA JONES‐LEWIS^1,2^, LORI BELL^1,2^, LARA G.B HEDLEY^1,2,3^, ADRIANA TOKICH^1,2,3^, ANUGRAH NAIR^1,2,3^, RAJEEV K. PATHAK^1,2,3,4^

#### 24.1.1 ^1^Canberra Heart Rhythm, Canberra, Australia,^2^Canberra Heart Rhythm Foundation, Canberra, Australia,^3^Australian National University, Canberra, Australia,^4^University of Canberra, Canberra, Australia

**Introduction:** Left bundle branch area pacing (LBBAP) has shown to be safe and effective, observing low and stable pacing thresholds. We aimed to prospectively assess the efficacy, safety, and long term outcomes of LBBAP.

**Methods:** In this cohort study, consecutive patients requiring pacing therapy were enrolled. Permanent LBBAP leads were implanted if the capture threshold was <2.5V/0.5ms. Established criteria was used to confirm left bundle branch (LBB) capture including pacing stimulus‐left ventricular activation time (S‐LVAT) in precordial lead V6 and V6‐V1 interpeak interval.

**Results:** In 330 enrolled patients (68±11 years of age, 63% male), LBB capture was acutely achieved in 317 (96%) patients, and permanent LBBAP in 307 (93%) patients. 23 (7%) patients did not receive permanent LBBAP, due to no LBB capture (n=11), high LBB capture thresholds (n=90) or fixation failure (n=2). The mean follow up period was 48±4 months.LBB capture threshold remained stable with acute threshold of 1.36±0.72V/0.5ms to 2.2±0.38V/0.5ms at 6 year follow up. S‐LVAT is 71.8±20.4ms. V6‐V1 interpeak interval was 53.2±11.3ms. Mean percentage RV pacing was 40±3%. Mean paced QRS duration at implant was 95±7ms and 103±10ms at final follow up. RV Lead Impedance was 461ohms at baseline and remained stable at 452ohms. Measured R‐Wave was 7.62ms at baseline and 7.83ms at final follow up.Mean left ventricular (LV) ejection fraction at baseline was 52.6±5%, which remained stable at 54.6±1.5% at final follow up. At baseline, left atrial indexed volume was 35±2.3mL/m^2^ and remained stable at 32±1.4mL/m^2^. LV end diastolic volume was 102.6±9.8mL/m^2^ at baseline and improved to 99.3±15.8mL/m^2^ at final follow up. No patient had worsening of RV function, mitral or tricuspid regurgitation.

**Conclusions:** This prospective cohort study suggests that left bundle branch area pacing is feasible, safe and effective with high success and low complication rates during long term follow up. Therefore, left bundle branch area pacing appears a reliable method for physiological pacing in patients with bradycardia indication.

## 25 INITIAL EXPERIENCE OF LEFT BUNDLE BRANCH AREA PACING IN CHILDREN

### 25.1 **YERLAN TURUBAYEV**, ABAY BAKYTZHANULY, SERIK BAGIBAYEV, ZHANDOS YESSILBAYEV, SARDOR YULDASHOV, TATYANA IVANOVA‐RAZUMOVA, OMIRBEK NURALINOV

#### 25.1.1 University Medical Center CF, Astana, Kazakhstan

**Introduction:** Conventional right ventricle pacing may cause pacing‐induced cardiomyopathy due to non‐physiological propagation of electrical activity. Electrical and mechanical dyssynchrony caused by chronic right ventricle pacing may disrupt contraction pattern. Conduction system pacing ‐ novel pacing modality which rapidly develops during the last years and nowadays it used as a first‐line approach in adults. Nowadays only limited data are available in conduction system pacing in pediatric population. In this observational, non‐randomized study we present initial experience of LBBAP in children to evaluate safety and feasibility of lbbap in pediatric patients.

**Methods:** Inclusion criteria ‐ patients under 18 years old with complete heart block and symptomatic bradycardia. We collected baseline data, previous medical history, pre‐procedural ECG and TTE, pacing parameters and procedural outcomes. mean follow‐up ‐ 6 months. Total 11 patients underwent implantation of LBBAP pacemaker. Mean age was 13.5±3.7 years and weight 47.6±18.6 kg. In all patients indication for pacing was symptomatic bradycardia due to high degree AV block.

**Results:** LBBAP was successfully achieved in all patients resulting narrow QRS complex and RBBB QRS morphology. Peak left ventricular activation time (pLVAT) (62.5±4.1ms), V6‐V1 (43.5±2.5ms) interpeak, presence of left bundle and fascicular potential (7 patients), transition from non‐selective to selective capture were recorded during the implantation. Mean paced QRS duration was 117.2±8.1 ms. Pacing thresholds, impedance remains stable (0.75±0.25V, and 615±50 Ohm). There were no major complications during implanting process and follow‐up.

**Conclusions:** LBBAP in pediatric patients appears as feasible and safe procedure, resulting narrow QRS, preserving ventricular synchrony with stable electrical parameters. Due to small cohort of patients, further collaborative large studies are needed to evaluate long‐term outcomes.

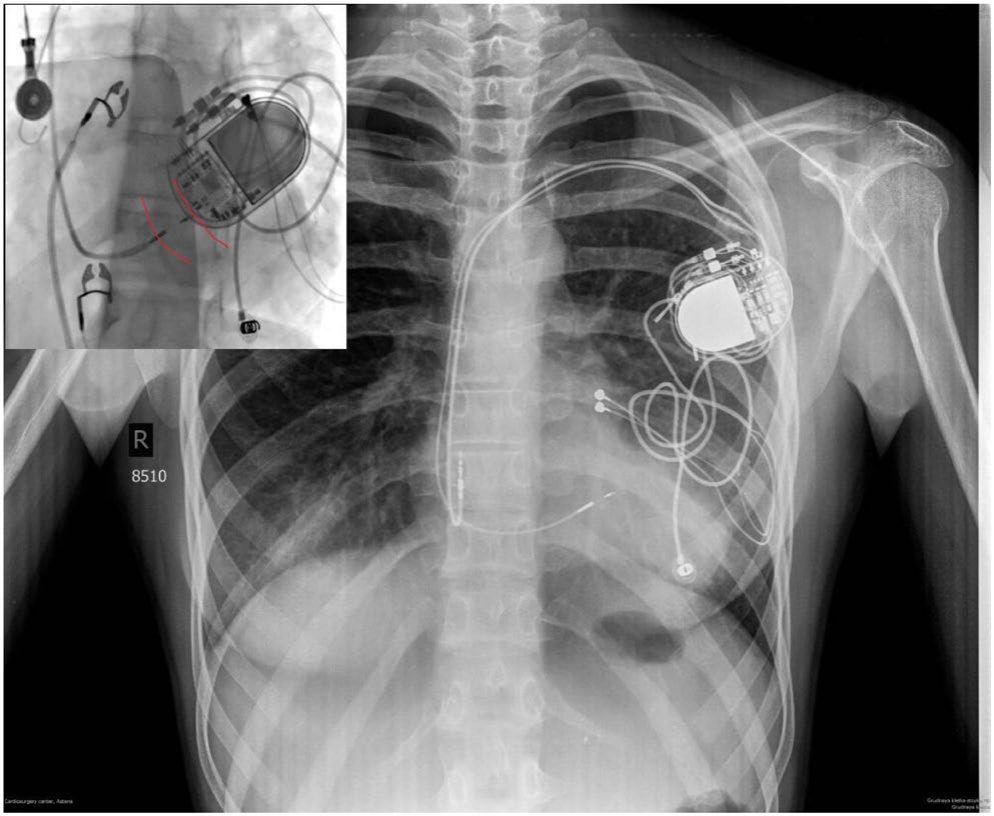

## 26 COMPARATIVE EFFICACY OF CRYOABLATION CATHETER TIP SIZES VS IRRIGATED AND NON‐IRRIGATED RADIO FREQUENCY ABLATION IN THE MANAGEMENT OF ATRIOVENTRICULAR NODAL REENTRY TACHYCARDIA: A SYSTEMATIC REVIEW AND NETWORK META‐ANALYSIS

### 26.1 DERREN RAMPENGAN^1^, MELISSA ARIYANTO^2^, NATHANAEL TJIPTA^2^, KETUT GANESHA PUTRA ANUGRAHA^2^, **SEBASTIAN WILLYANTO** ^3^, IMKE PULING^3^, NYOMAN GIRI^3^

#### 26.1.1 ^1^Universitas Sam Ratulangi, Manado, Indonesia,^2^Universitas Airlangga, Surabaya, Indonesia,^3^Universitas Brawijaya, Malang, Indonesia

**Introduction:** Atrioventricular nodal reentrant tachycardia (AVNRT) is the most prevalent type of supraventricular tachycardia that can manifest at any age. Catheter ablation is a successful and widely used approach as first line treatment for AVNRT. However, there is still debate about the choice of the most optimal energy. Radiofrequency ablation (RFA) is highly effective but carries a risk of atrioventricular block (AVB). On the other hand, cryoablation (CBA) is well known for its safety but its effectiveness is arguably lower. Moreover, different types of tip sizes used in cryoablation show a variative outcome. Same case applies to the use of irrigation in radiofrequency. Thus, the purpose of this study is to assess the efficacy between different tip sizes of CBA and RFA for the treatment of AVNRT.

**Methods:** A systematic literature search was conducted based on the PRISMA NMA Checklist of Items in six databases. Network meta‐analysis was done using RStudio. We also performed surface under cumulative ranking curve (SUCRA), subgroup, and meta‐regression analysis.

**Results:** The network meta‐analysis found that non‐irrigated RFA has the lowest risk of developing AVB (OR = 0.51; 95% CI = 0.04 ‐ 7.32) with a downfall of highest recurrence rate (OR = 0.76; 95% CI = 0.29 ‐ 2.02), lowest energy of application (OR = ‐0.50; 95% CI = ‐11.44 ‐ 10.44), and shortest procedural time (OR = ‐3.54; 95% CI = ‐7.72 ‐ 0.64). On the other hand, CBA 4 mm has the shortest fluoroscopy time (OR = 3.57; 95% CI = 2.27 ‐ 4.87) and CBA 8 mm is associated with the highest success rate (OR = 1.89; 95% CI = 0.84 ‐ 4.23).

**Conclusions:** When it comes to efficacy, non‐irrigated RFA is the most effective method with lowest risk of recurrence rate and fastest procedural time and operation. With the lowest energy application and the lowest risk of AVB, RFA is deemed preferable in terms of safety, although fluoroscopy duration is second only to CBA 4 mm.